# Improvement of *Panax notoginseng* saponin accumulation triggered by methyl jasmonate under arbuscular mycorrhizal fungi

**DOI:** 10.3389/fpls.2024.1360919

**Published:** 2024-03-13

**Authors:** Hong-Yang Dai, Xing-Kai Zhang, Yue Bi, Di Chen, Xian-Nv Long, Yue Wu, Guan-Hua Cao, Sen He

**Affiliations:** ^1^ School of Chinese Materia Medica, Yunnan University of Chinese Medicine, Yunnan, Kunming, China; ^2^ Kunming Lancang-Mekong Regional R&D Central for the Development Utilization of Traditional Medicine Resources, Yunnan University of Chinese Medicine, Yunnan, Kunming, China; ^3^ College of Pharmacy, Zhaotong Health Vocational College, Yunnan, Zhaotong, China

**Keywords:** *Panax notoginseng*, arbuscular mycorrhizal fungi (AMF), Methyl jasmonate (MeJA), notoginsenoside, key enzymes, transcriptome sequencing

## Abstract

*Panax notoginseng* is a highly valued perennial medicinal herb plant in Yunnan Province, China, and the taproots are the main medicinal parts that are rich in active substances of *P. notoginseng* saponins. The main purpose of this study is to uncover the physiological and molecular mechanism of *Panax notoginseng* saponin accumulation triggered by methyl jasmonate (MeJA) under arbuscular mycorrhizal fungi (AMF) by determining physiological indices, high-throughput sequencing and correlation analysis. Physiological results showed that the biomass and saponin contents of *P. notoginseng*, the concentrations of jasmonic acids (JAs) and the key enzyme activities involved in notoginsenoside biosynthesis significantly increased under AMF or MeJA, but the interactive treatment of AMF and MeJA weakened the effect of AMF, suggesting that a high concentration of endogenous JA have inhibitory effect. Transcriptome sequencing results indicated that differential expressed genes (DEGs) involved in notoginsenoside and JA biosynthesis were significantly enriched in response to AMF induction, e.g., upregulated genes of diphosphocytidyl-2-C-methyl-d-erythritol kinases (*ISPEs*), cytochrome P450 monooxygenases (*CYP450s*)*_*and glycosyltransferases (*GTs*), while treatments AMF-MeJA and salicylhydroxamic acid (SHAM) decreased the abundance of these DEGs. Interestingly, a high correlation presented between any two of saponin contents, key enzyme activities and expression levels of DEGs. Taken together, the inoculation of AMF can improve the growth and saponin accumulation of *P. notoginseng* by strengthening the activities of key enzymes and the expression levels of encoding genes, in which the JA regulatory pathway is a key link. This study provides references for implementing ecological planting of *P. notoginseng*, improving saponin accumulation and illustrating the biosynthesis mechanism.

## 1 Introduction

*Panax notoginseng* (Burk.) F. H. Chen, belonging to the genus *Panax*, family Araliaceae, is a highly valued perennial medicinal herb plant that is widely distributed in Yunnan Province, China, of which the taproots are the main medicinal parts. Saponins are the main active substances and have remarkable pharmacological value and biological activities in animal experiments or clinical treatment, e.g., cardioprotective effects, protection against cerebrovascular injury, neuroprotective effects, anti-tumor, anti-inflammatory activities, hemostasis and anti-coagulation (Liu et al., 2020; Jiang et al., 2021; Cheng et al., 2023; Xia Z. et al., 2023).

*P. notoginseng* saponins (PNSs) are mainly composed of notoginsenoside R1, ginsenosides Rg1, Rb1, Rd and Re, and some rare saponins, of which these monomeric saponins are dammarane-type tetracyclic triterpenoids. PNSs are synthesized either in the cytosol through the mevalonate (MVA) pathway, or in plastids through the methylerythritol phosphate (MEP) pathway (Yang et al., 2018; Hu et al., 2022), both of which are derived from the universal five-carbon precursors, isopentenyl diphosphate (IPP) and dimethylallyl diphosphate (DMAPP), respectively. Many key enzymes, e.g., 3-hydroxy-3-methyl-glutaryl-CoA reductase (HMGR), squalene synthase (SS), squalene epoxidase (SE), dammarenediol synthase (DS), farnesyl pyrophosphate (FPP), cytochrome P450 monooxygenase (CYP450) and glycosyltransferase (GT), are involved in this biosynthetic pathway (Yang et al., 2018; Hu et al., 2022), of which the SS, SE, CYP450 and GT were identified as main key enzymes in plants that are essential to the formation and diversification of PNSs (Cheng et al., 2020; Huang et al., 2023; Wang Q. et al., 2023). SS catalyzes two FPP molecules into a C30 isoprenoid squalene, then SE oxidizes the squalene to 2,3-oxidosqualene, the precursor for triterpene biosynthesis (Bai et al., 2021; Cun et al., 2024). CYP450s catalyzing a carboxyl group at C-28 and hydroxyl groups at C-2β, C-16α, C-23 and C-24 form multiple sapogenins (Tamura et al., 2017; Pan et al., 2023). GTs that can glycosylate the sapogenins at the C-3 and C-28 positions are predicted to form monodesmosidic or bisdesmosidic saponins with specific structures and activities (Cheng et al., 2020). However, notoginsenoside biosynthesis and accumulation mechanisms in response to the induction of biological factors and environmental factors such as arbuscular mycorrhizal fungi (AMF) are poorly understood.

AMF are a class of ancient fungi that can form a mutualistic symbiosis with over 80% of plant species roots in natural and agricultural systems and are mainly classified as Glomeromycota, including Diversisporales, Archaeosporales, Glomerales and Paraglomerales (Camargo-Ricalde, 2002; Malar et al., 2022; Fan et al., 2023). Increasing evidence has shown that AMF play key roles in the growth and accumulation of secondary metabolites in medicinal plants (Kumar et al., 2021; Ran et al., 2022; Zhao et al., 2022; Yuan et al., 2023; Cao et al., 2023b). The contents of many active substances increased under the inoculation of AMF, e.g., artemisinin from *Artemisia annua* L (Mandal et al., 2015; Domokos et al., 2018), glycyrrhizin and liquiritin from *Glycyrrhiza uralensis* Fisch (Xie et al., 2018, 2019), aloe emodin from *Polygonum cuspidatum* Sieb. Et Zucc (Sun et al., 2022). However, the effect of different AMF species on the accumulation of plant metabolites varies greatly (Majewska et al., 2017; Ren et al., 2021; Ran et al., 2021a; Marro et al., 2022). Therefore, choose suitable AMF agents is a key step to improve the accumulation of active substances within plants.

Studies have shown that the mechanisms of growth promotion and metabolite accumulation in plants mediated by AMF are mainly related to the improvement of the host plant’s uptake of phosphorus and other nutrient elements, the enhancement of photosynthesis, the upregulated expression levels of key enzyme-encoding genes, and the regulation of phytohormones (Lin et al., 2017; Majewska et al., 2017; Ran et al., 2021a). It was shown that jasmonates (JAs) and abscisic acid (ABA) affect mycorrhization, particularly in JAs, possibly in multiple ways (Hause et al., 2007; Ludwig-Müller, 2010; Foo et al., 2013). Jasmonates (JAs) are a class of endogenous phytohormones and are involved in growth regulation within plants, system defense and active substance biosynthesis, in which methyl jasmonate (MeJA) is a derivative of JA produced by the α-linolenic acid (18:3) pathway and hexadecanotrienoic acid (16:3) pathway (Kazan, 2015; Chini et al., 2018; Li et al., 2020). Allene oxide cyclase (AOC) and lipoxygenase (LOX) are two key enzymes that produces 12-oxo-phytodienoic acid (OPDA) with 12,13(S)-epoxy-octadecatrienoic acid and initiates hydroperoxidation of polyunsaturated fatty acids, respectively, then establishes the stereochemical configuration of naturally occurring JA (Li et al., 2020; Viswanath et al., 2020; Yang et al., 2023).

Many studies have shown that MeJA participates in the regulation of triterpenoid biosynthesis in medicinal plants, e.g., *Saponaria vaccaria* L., *Talinum paniculatum* (Jacq.) Gaertn., *Betula platyphylla* Sukaczev, *Ocimum basilicum* L., and *Withania somnifera* (L.) Dunal (Misra et al., 2017; Faizal and Sari, 2019; Sharma et al., 2019; Yin et al., 2020; Chen et al., 2023). In this process, the activities of key enzymes involved in triterpenoid biosynthesis and the expression levels of related genes were affected by the MeJA concentration (Yin et al., 2020; Dinday and Ghosh, 2023; Li et al., 2023; Ning et al., 2023; Xia Q. et al., 2023).

However, the relationship among AMF colonization, saponin accumulation and MeJA concentration, particularly the effect of AMF colonization rates on the accumulation of PNSs and the role of MeJA, is unclear. In this study, we highlighted the physiological and molecular mechanism of PNS accumulation induced by AMF using an intermediate role of MeJA. This research has important theoretical and practical significance for implementing the ecological planting of *P. notoginseng*, improving the accumulation of saponins, and clarifying the synthesis mechanism of PNSs.

## 2 Materials and methods

### 2.1 Materials and group design

Plant materials: The annual seedlings of *P. notoginseng* used for treatments were bought from Miaoxiang Sanqi standard plantation that located in Zhuang-miao Autonomous Prefecture of Wenshan Yunnan Province, China. These seedlings had homogeneity in growth status and biomass. AMF strains: Spores of *Glomus intraradices* and *G. etunicatum* mixed in the soil were purchased from the Institute of Root Biology, Wuhan Yangtze University. After morphological identification, the AMF spore soil was mixed into the sterilized soil culture substrate at a ratio of 1:20, and then the seeds of maize and clover were planted in the soil, of which the root systems were employed for spore propagation in semi closed conditions.

Experimental groups: In total, five treatment groups treated with one or two factors of AMF, MeJA and SHAM, including AMF, AMF-MeJA, AMF-SHAM, MeJA and SHAM, and one blank control group (CK) were set up, among which the AMF was a long-term treatment group for five months, and the MeJA and SHAM were short treatments for one month before sampling. The *P. notoginseng* seedlings were randomly assigned to groups, and each group contained more than 30 plants. The cultivation substrate used in this experiment was composed of nutrient soil and vermiculite (18:1), which were mixed evenly and sterilized with intermittent humid heat (121 °C, 2 h for twice). After drying, the sterilized cultivation substrate was subpackaged into clean pots, and each pot contained 1.5 kg mixed soil. Before planting, the *P. notoginseng* seedlings described above were rinsed with purified water to remove the attached soil and sprayed with benomyl to inhibit the indigenous strains. Three *P. notoginseng* seedlings were planted into each pot, and 15-20 g AMF soil containing about 100 spores (*G. intraradices*:*G. etunicatum* = 1:1) was placed 1~2 cm around roots. After 5 months, *P. notoginseng* seedlings were treated three times by irrigating roots with 20 mL of 100 µmol/L MeJA or SHAM every time, in which SHAM is a specific inhibitor of LOX in the biosynthetic pathway of JA.

*P. notoginseng* has strict requirements for the environmental conditions. The seedlings were planted in a greenhouse at 25 ± 3°C and 60 ± 5% relative humidity, avoiding direct sunlight and water accumulation and maintaining ventilation. After six months, the fresh roots were harvested and used for RNA extraction, transcriptome sequencing and the determination of PNS contents and activities of key enzymes involved in the biosynthesis of saponins and JAs. The remaining samples were stored at -80°C.

### 2.2 Determination of biomass and notoginsenoside contents

The biomass of *P. notoginseng*, including plant height, total fresh weight and fresh root weight, were measured directly, and the notoginsenoside contents were determined using high performance liquid chromatography (HPLC). Dried sample powder of 0.6 g was placed in 25 mL volumetric bottle, with 70% methanol at constant volume to the scale. Saponins were extracted for 45 min using an ultrasonic extraction method, then the solution was continuously filtered with 0.45 μm filter paper and 0.22µm filter membrane, respectively (Cao et al., 2023a). A binary gradient elution system consisted of acetonitrile (A) and water (B), and the separation was achieved using the program listed in **Supplementary Table 1**. The parameters of flow rate, injection volume, detection wavelength and column temperature were 1 mL/min, 20 μL, 203 nm and 30°C, respectively. Linear regression equations of ginsenosides Rg1, Rb1, Rd, Re and notoginsenoside R1 were generated and are listed in **Supplementary Table 2**.

### 2.3 Determination of endogenous phytohormone concentrations

The concentrations of five phytohormones including JA, MeJA, jasmonoyl-L-isoleucine (JA-Ile), 12-oxophytodienoic acid (OPDA) and salicylic acid (SA) were determined using an ultra-performance liquid chromatography (UPLC) series SCIEX-6500Q trap mass spectrometer (MS/MS). Approximately 50 mg of fresh root samples were ground to powder with liquid nitrogen, and then added 10 µL of 100 ng/mL corresponding internal standard material and 1 mL mixed extractant of methanol/water/formic acid (15:4:1), followed by centrifugation, concentration and redissolution (Floková et al., 2014; Li et al., 2016). Chromatographic separation of five types of phytohormones was performed using a Waters ACQUITY UPLC HSS T3 C18 column (1.8 µm, 100 mm×2.1 mm). Phase A (water/0.4% acetic acid solution) and phase B (acetonitrile/0.4% acetic acid solution) of LC–MS/MS were utilized to conduct gradient elution at a constant flow rate of 0.35 mL/min at 40°C, and the injection volume was 2 µL (Cai et al., 2014; Niu et al., 2014; Xiao et al., 2018). The elution program was performed according to **Supplementary Table 3**. In this experiment, the electrospray ionization (ESI) temperature was set at 550°C, and the mass spectrum voltage was set at 5,500 V in positive ion mode and -4,500V in negative ion mode. The curtain gas (CUR) was set at 35 psi (Pan et al., 2010; Cui et al., 2015; Šimura et al., 2018). Linear regression equations of JA, MeJA, JA-Ile, OPDA and SA were generated and are listed in **Supplementary Table 4**.

### 2.4 Determination of enzyme activity

The activities of key enzymes involved in notoginsenoside biosynthesis, including SS, SE, CYP450 and GT, were determined using plant ELISA kits (SS, SE and CYP450: 96T, Jiangsu Meimian Industrial Co., Ltd, China; GT: 96T, Quanzhou jiubang Biotechnology Co., Ltd, China) (Yin et al., 2017; Wang L. et al., 2021; Zhang et al., 2021). The activities AOC and LOX are two main key enzymes that are responsible for the biosynthesis of JA, and their activities were measured according to the instructions of the plant allene oxide cyclase enzyme activity assay ELISA Kit (96T, Quanzhou Jiubang Biotechnology Co., Ltd, China) and plant lipoxygenase enzyme activity assay EELISA Kit (96T, Quanzhou Jiubang Biotechnology Co., Ltd, China), respectively (Qiu et al., 2020).

### 2.5 RNA extraction, library construction and sequencing

Three replicate fresh root samples were randomly selected from each group, stored in liquid nitrogen and then sent to MetWare (Wuhan Metwell Biotechnology Co., Ltd., China) for RNA sequencing (RNA-Seq). When the integrity, purity and concentration of RNA were qualified, double-stranded cDNA could be synthesized using kits and then sequenced using the Illumina HiSeq platform.

### 2.6 *De novo* transcriptome assembly and functional annotation

The raw data were quality controlled by fastp software (Chen et al., 2018). After sequencing data filtering, GC content distribution checking and sequencing error rate checking, clean reads were acquired for *de novo* assembly. Original RNA-sequence data were deposited in the National Center for Biotechnology Information (NCBI) with the accession number PRJNA953146. All unigenes were searched against the NCBI protein nonredundant (NR), Karyotic Orthologous Groups (KOG), Kyoto Encyclopedia of Genes and Genomes (KEGG) and Gene Ontology (GO) databases using DIAMOND BLASTX with a threshold of E < 1.0E-5 to identify the proteins whose sequences were most similar to those of the given transcripts to retrieve their functional annotations (Buchfink et al., 2015). Functional annotation of proteins was carried out by searching against the NCBI non-redundant nucleotide (Nt) database using BLASTn algorithms with a threshold of E < 1.0E-5.

### 2.7 Differential expression analysis and functional enrichment

The expression level of each transcript was quantified using the RSEM program (Li and Dewey, 2011; Langmead and Salzberg, 2012). DESeq2 and edgeR were used for differential expression analysis of samples, in which |log_2_^(Fold Change)^| ≧ 1 and false discovery rate (FDR) < 0.05 were considered to be significantly differentially expressed (Robinson et al., 2010; Love et al., 2014; Varet et al., 2016). DESeq2 and edgeR are implemented as a package for the R statistical environment, and the count matrix and metadata are stored in an S4 class derived from the *SummarizedExperiment* class of the *GenomicRanges* package (http://www.bioconductor.org/packages/release/bioc/html/DESeq2.html) and Bioconductor project (http://bioconductor.org/packages/RNAseq123), respectively (Lawrence et al., 2013; Love et al., 2014). The GO and KEGG were employed to analyze the functional enrichment and metabolic pathways of *P. notoginseng* transcriptomic data, and a corrected P value < 0.05 indicated that the enrichment was more significant. The transcripts per million (TPM) reads of these unigenes were obtained from RNA-seq data, and the log_2_^(TPM)^ values of the differential expressed genes (DEGs) were repeatedly calculated based on three biological replicates. The online tool SangerBox 3.0 (http://vip.sangerbox.com/home.html) was used to generate heatmaps of DEGs involved in notoginsenoside (Shen et al., 2022).

### 2.8 Validation of RNA-seq data by qRT-PCR

A quantitative real-time PCR (qPCR) validation assay was carried out according to the description of Cao et al. (2023a), and 10 target genes and *β-actin* (reference gene) were used to validate the DEG results of Illumina. All the primers used for qPCR are listed in **Supplementary Table 5**, and the qPCR was carried out using TB Green^®^ Premix Ex Taq™ II (Takara) on a LightCycler^®^96 real-time PCR system (Roche, Switzerland). The data were processed using Q=2^-ΔΔCT^ to obtain the relative expression level of each gene, in which Q means the relative expression level.

### 2.9 Statistical analysis

All data were processed and analyzed statistically with Microsoft Excel 2010 and SPSS 26.0. Assumptions of normality and homogeneity of variance were tested prior to all statistical tests. The significant differences were tested with one-way analysis of variance (ANOVA), followed by Tukey tests at the level of 0.05. Origin 2021 and R 4.3.2 were used for the data visualization. Standard deviation (SD) were used for error bars, n≧3. The biomass, contents of saponins, activities of key enzymes and concentrations of JAs were presented as the mean ± SD, n≧3. The correlation among DEGs, saponin and JAs’ contents and key enzyme activities were accomplished using the Mantel test with linkET R package (https://github.com/Hy4m/linKET) (Huang, 2021). Pearson analysis were used to determine the correlation of expression levels of DEGs and activities of key enzymes, and the threshold was set as r > 0.6 (Wang H.R. et al., 2023).

## 3 Results

### 3.1 Biomass and notoginsenoside contents of *P. notoginseng*

**Figure 1:**
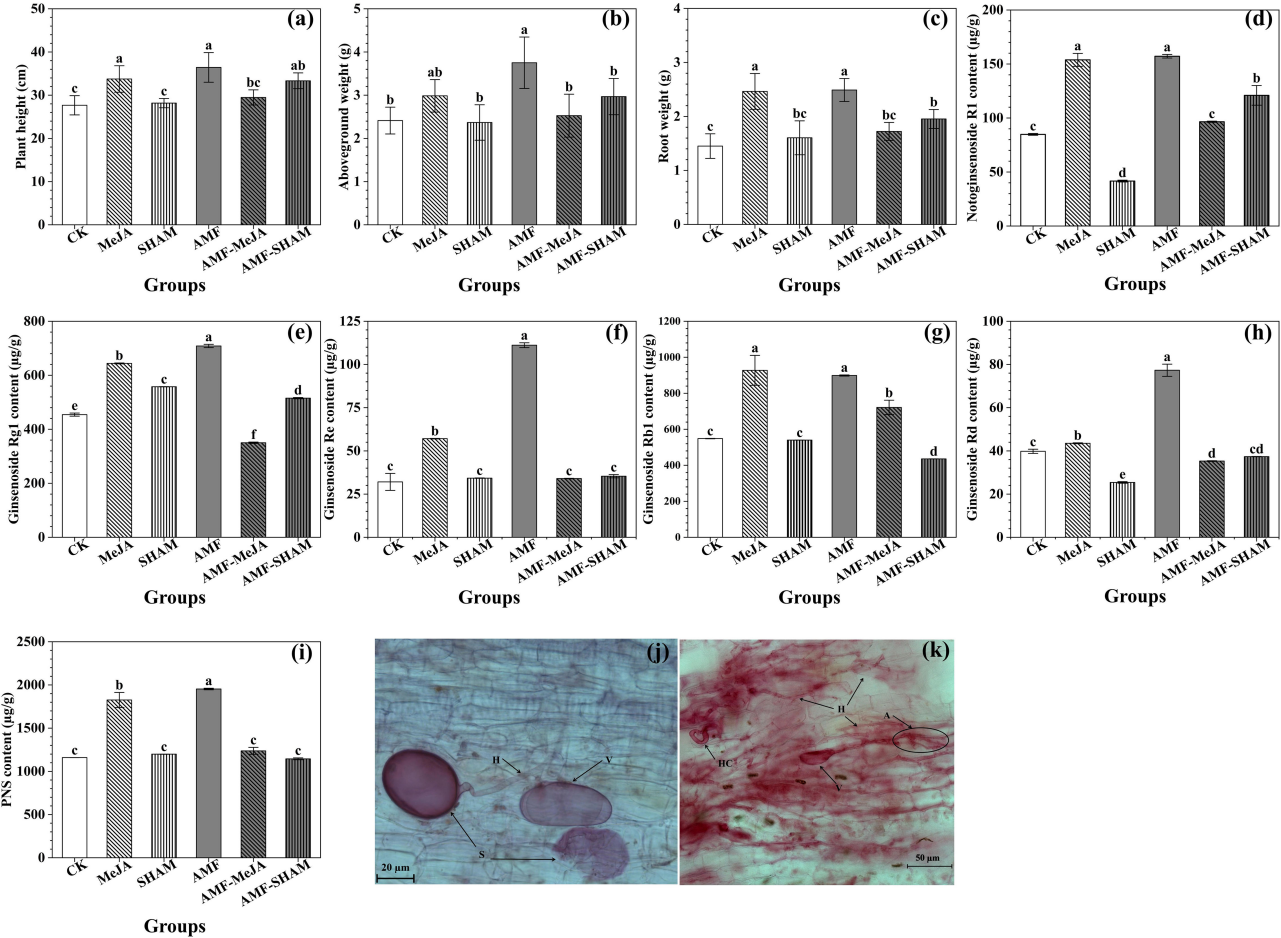
Effects of AMF, MeJA and SHAM on *P. notoginseng* growth and notoginsenoside contents. **(A)** Plant weight. **(B)** Aboveground weight. **(C)** Root weight. **(D)** Notoginsenoside R1. **(E)** Ginsenoside Rg1. **(F)** Ginsenoside Re. **(G)** Ginsenoside Rb1 **(H)** Ginsenoside Rd. **(I)** *P. notoginseng* saponins (PNS). **(J, K)** Typical structure of AMF and their communities colonized in *P. notoginseng* fibrous roots; A: arbuscule H: hypha; HC: hyphae circle; V: vesicles; S: spore. A total of five treatment groups treated with AMF, AMF-MeJA, AMF-SHAM, MeJA and SHAM, respectively, and one blank control group (CK). AMF and exogenous MeJA significantly promoted the growth and saponin accumulation of *P. notoginseng*, particular in AMF. However, the addition of SHAM had little effect on the biomass and saponin contents, except for saponin Rg1. Different lowercase letters represent significant differences among different treatment groups (*P* < 0.05).

As shown in **Figure 1**, AMF inoculation, as well as the addition of exogenous MeJA, significantly promoted the growth of *P. notoginseng*, of which the plant height and the fresh weight of roots and aboveground parts increased by 31.69/21.99, 55.46/23.69 and 71.53/69.69% (AMF/MeJA), respectively, compared with the CK group, directly indicating that the promoting effect of AMF was higher than that of MeJA. The biomass of *P. notoginseng* was not significantly reinforced under the superimposed treatment of AMF and MeJA. Compared with AMF, the addition of SHAM (AMF-SHAM) significantly decreased the promoting effect of AMF (P < 0.05), and the inhibitory rates to plant height and the fresh weight of roots and aboveground parts were 8.51, 20.88 and 21.55%, respectively.

Compared to CK, the contents of notoginsenoside R1, ginsenoside Rg1, Re, Rb1, Rd and PNSs significantly increased by 1.85, 1.56, 3.47, 1.64, 1.94 and 1.68 times, respectively, under the AMF treatment (P < 0.05). The addition of exogenous MeJA also significantly increased the contents of five monomeric saponins and PNSs. However, the saponin contents of *P. notoginseng* in the AMF-MeJA and SHAM groups were significantly lower than that of AMF, and the decreasing amplitudes of notoginsenoside R1, ginsenoside Rg1, Re, Rb1, Rd and PNSs were 38.57/73.51, 50.58/21.29, 69.42/69.21, 19.73/39.94, 54.37/67.17 and 36.64/38.62% (AMF-MeJA/SHAM), respectively, suggesting that the biosynthesis and accumulation of notoginsenosides were largely affected by AMF colonization and that the addition of MeJA participated in the relevant regulatory process. In addition, the supplementation with SHAM could significantly increase the content of ginsenoside Rg1, but it was lower than the promoting effect of AMF and MeJA.

### 3.2 The activities of key enzymes involved in notoginsenoside biosynthesis

**Figure 2:**
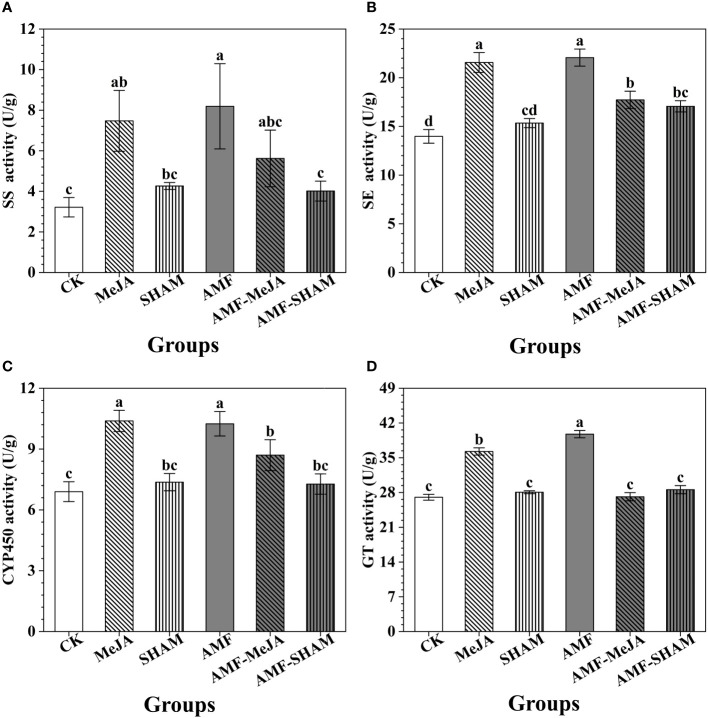
Effects of AMF, MeJA and SHAM on the activities of key enzymes involved in notoginsenoside biosynthesis. **(A)** The activity of squalene synthases (SS). **(B)** The activity of squalene epoxidases (SE). **(C)** The activity of cytochrome p450 monooxygenases (CYP450). **(D)** The activity of glycosyltransferases (GT). The addition of AMF and MeJA significantly improved the activities of key enzymes SS, SE, CYP450 and GT. However, the supplementation with SHAM significantly weakened the roles of AMF in enzyme activity promotion. Different lowercase letters represent significant differences among different treatment groups (*P* < 0.05).

The activities of key enzymes SS, SE, CYP450 and GT increased by 2.55-, 1.58-, 1.49- and 1.47-fold, respectively, under AMF inoculation (**Figure 2**). Similar changes were also observed in the MeJA group, in which the activities of the key enzymes SS, SE, CYP450 and GT increased by 2.32-, 1.54-, 1.51- and 1.34-fold, respectively. In general, the activities of SE, CYP450 and GT in the AMF-MeJA group were significantly lower than those in AMF and MeJA groups, but higher than those in the CK group. The addition of SHAM improved the activities of key enzymes, but it can weaken the roles of AMF in activity promotion. Compared to the AMF group, the activities of the key enzymes SS, SE, CYP450 and GT in SHAM decreased by 51.02, 22.67, 29.02 and 28.13%, respectively (**Figure 2**).

### 3.3 The concentrations of the endogenous plant hormones JAs and SA

**Figure 3:**
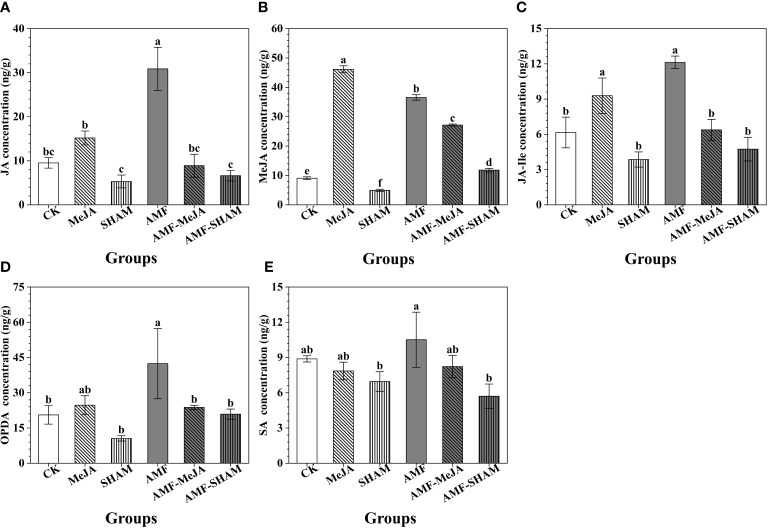
Effects of AMF, MeJA and SHAM on the concentrations of endogenous JAs and SA in *P. notoginseng* fibrous roots. **(A)** The concentration of JA. **(B)** The concentration of MeJA. **(C)** The concentration of JA-Ile. **(D)** The concentration of OPDA. **(E)** The concentration of SA. The concentrations of endogenous JAs (JA, MeJA, JA-Ile and OPDA) and SA significantly increased under AMF treatment but the interaction with MeJA or SHAM can weaken the promotion effect. The addition of MeJA increased the concentrations of JAs. Different lowercase letters represent significant differences among different treatment groups (P < 0.05).

As shown in **Figure 3**, the concentrations of endogenous JAs, including JA, MeJA, JA-Ile and OPDA, were 30.86, 36.56, 12.13 and 42.39 ng/g, respectively, under inoculation with AMF and significantly increased by 3.24-, 4.04-, 1.97-, 2.06- and 1.25-fold compared to the CK group. This result suggested that the colonization of AMF participated in the biosynthesis and accumulation of endogenous JAs in the fibrous roots of *P. notoginseng*. The addition of exogenous MeJA also caused an increase in endogenous JAs, particularly JA, MeJA and JA-Ile, suggesting that short-time treatment with MeJA disturbed the metabolism of endogenous JAs. The concentrations of JA, JA-Ile and OPDA in AMF-MeJA group were significantly lower than those in the AMF group and close to those in the CK group, speculating that the addition of MeJA inhibited the regulatory effect of AMF on the JAs. However, as a specific inhibitor to JA biosynthesis, the addition of SHAM decreased the concentrations of JA, MeJA, JA-Ile and OPDA, of which the concentrations were significantly lower than those of the AMF and MeJA groups, particularly in MeJA. Compared to CK, the SA concentration decreased in all treatment groups except for AMF.

### 3.4 The activities of key enzymes AOC and LOX involved in JA biosynthesis

**Figure 4:**
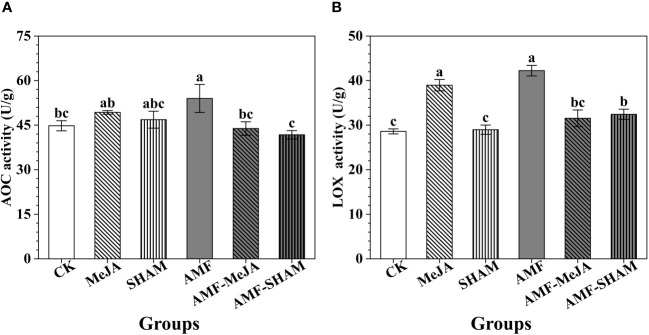
Effects of AMF, MeJA and SHAM on the key enzyme activities of AOC and LOX that involved in JA biosynthesis. **(A)** The activity of AOC. **(B)** The activity of LOX. The activities of AOC and LOX in *P. notoginseng* roots under inoculation with AMF were greater than those of the other groups. The addition of MeJA increased the activities of AOC and LOX but the interaction of AMF and MeJA decreased the activities, suggesting that the addition of exogenous MeJA can reduce the role of AMF in regulating JA biosynthesis. The addition of SHAM had no significant effect on AOC and LOX activities. Different lowercase letters represent significant differences among different treatment groups (P < 0.05).

AOC and LOX are two key enzymes that continuously catalyze the substrates hexadecatrienoic acid and α-linolenic acid in chloroplasts, which is a key step in JA biosynthesis. As shown in **Figure 4**, the activities of AOC and LOX in *P. notoginseng* roots under AMF were greater than those of the other groups. The addition of MeJA increased the activities of AOC and LOX, and a significant difference in LOX activity was observed between the MeJA and CK groups. Compared to the AMF group, the activities of AOC and LOX decreased significantly under AMF-MeJA, suggesting that the addition of exogenous MeJA can reduce the role of AMF in regulating JA biosynthesis. In addition, the short-term treatment with SHAM had no significant effect on AOC and LOX activities.

### 3.5 Transcriptome sequencing and assembly

The molecular mechanisms of notoginsenoside biosynthesis mediated by MeJA under the inoculation of AMF were determined by RNAseq analysis. Six transcriptomes were generated from the roots of groups AMF, MeJA, AMF-MeJA, SHAM, AMF-SHAM and CK, respectively. Eighteen transcriptomes from the six groups with three replicates yielded a total of 1,108,183,168 clean reads from 1,155,716,266 raw reads, and the clean data size of each transcriptome was larger than 8.46 Gb. The values of Q20 and Q30 for the above transcriptomes were in the ranges of 97.42~97.96% and 93.1~94.36%, respectively, and the overall sequencing error rate was 0.03% (**Supplementary Table 6**). Clean reads of 76.20~81.00% can be mapped to a single locus of the *P. notoginseng* genome with RSEM software (**Supplementary Table 6**). Pearson’s correlation coefficient showed that treatment groups of AMF, MeJA, SHAM, AMF-MeJA and AMF-SHAM had a strong correlation (R^2^ ≧ 0.75) between two duplicate samples. However, the correlation between CK and the other groups was relatively low, with a range of 0.53~0.88 (**Supplementary Figure 1**).

### 3.6 Screening and confirmation of DEGs and functional annotation

**Figure 5:**
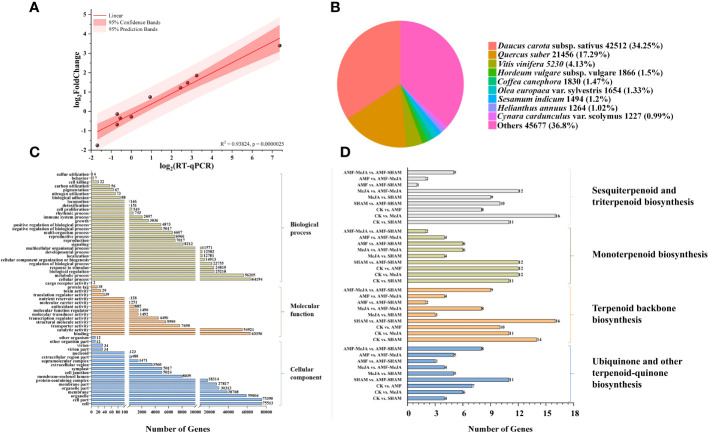
Confirmation of DEGs and functional annotation. **(A)** Validation of RNA-seq differentially expressed genes using qRT–PCR. The expression changes of target genes detected by RNA-seq were closely related to those determined by qRT–PCR with a correlation coefficient R^2^ of 0.93824, which confirmed the accuracy and reliability of the RNA-seq results. **(B)** Annotated species matching distribution in the *P. notoginseng* NR database. More than a third and close to a sixth of unigenes were homologous to the *D. carota* and *Q. suber*, respectively. **(C)** Gene Ontology classification of DEGs. A total of annotated unigenes (approximately 41.37%) of *P. notoginseng* with BLAST matches to known proteins were assigned to 3 main GO categories, including biological process (290,760, 37.27%), cellular component (348,933, 44.73%) and molecular function (140,390, 18.00%). **(D)** KEGG classification of DEGs involved in terpenoid biosynthesis. In total, 37, 45 and 40 DEGs that involved in terpenoid biosynthesis (triterpenoids, sesquiterpenoids, monoterpenoids and other terpenoids), were selected by the pairwise comparison of AMF vs. CK, MeJA vs. CK and SHAM vs. CK, respectively.

Compared to the CK group, a total of 3,553 DEGs were detected in the AMF group, including 1,461 (41.12%) and 2,092 (58.88%) upregulated and downregulated genes, respectively. Ten DEGs distributed in different families were selected for qRT–PCR validation, and the line correlation of DEGs in qRT–PCR and RNA-seq were compared by normalizing FPKM values with log_2_^(Fold_change)^. The results showed that the expression changes detected by RNA-seq were closely related to those determined by qRT–PCR, with a correlation coefficient R^2^ of 0.93824 (**Figure 5A**), which confirmed the accuracy and reliability of the RNA-seq results.

In total, 124,110, 94,304, 77,830, 124,298 and 84,972 unigenes were matched to existing gene models in the NR, KEGG, Pfam, Trembl and SwissProt protein databases, respectively (**Supplementary Table 7**). However, the match ratios were not as high as expected due to the absence of research on RNA-seq of plant fibrous roots, particularly in *P. notoginseng*. As illustrated in **Figure 5B**, 42,512, 21,456 and 5,130 unigenes in 124,110 were homologous to *Daucus carota* subsp. sativus, *Quercus suber* L. and *Vitis vinifera* L., respectively.

The results of GO classification showed that 106,540 annotated unigenes (approximately 41.37%) of *P. notoginseng* with BLAST matches to known proteins were assigned to 3 main GO categories, including biological process (290,760, 37.27%), cellular component (348,933, 44.73%) and molecular function (140,390, 18.00%). These GO terms were further subdivided into 60 subcategories, and gene-encoding “binding” and “catalytic activity” proteins accounted for a large part of the molecular function category, e.g., key enzymes for saponin synthesis (**Figure 5C**; **Supplementary Table 8**). The results of KEGG analysis showed that 94,304 unigenes (approximately 36.62%) were found to have significant matches in the KEGG pathway database (**Supplementary Table 9**). DEGs that are involved in metabolic pathways and biosynthesis of secondary metabolites were closely related to treatments such as AMF and MeJA, in which 37 (AMF vs. CK) and 45 (MeJA vs. CK) DEGs were involved in the biosynthesis of terpenoids, respectively, e.g., triterpenoids, sesquiterpenoids and monoterpenoids (**Figure 5D**). According to KEGG annotation, many unigenes in the items, e.g., lipid transport and metabolism, carbohydrate transport and metabolism, energy production and conversion and signal transduction and mechanisms, were found to be closely related to the substance and energy exchange of the AMF-*P. notoginseng* symbiotic system and signal pathways mediated by plant hormones (**Supplementary Figure 2**; **Supplementary Table 10**).

### 3.7 Identification of DEGs involved in the biosynthesis of notoginsenosides and JAs

**Figure 6:**
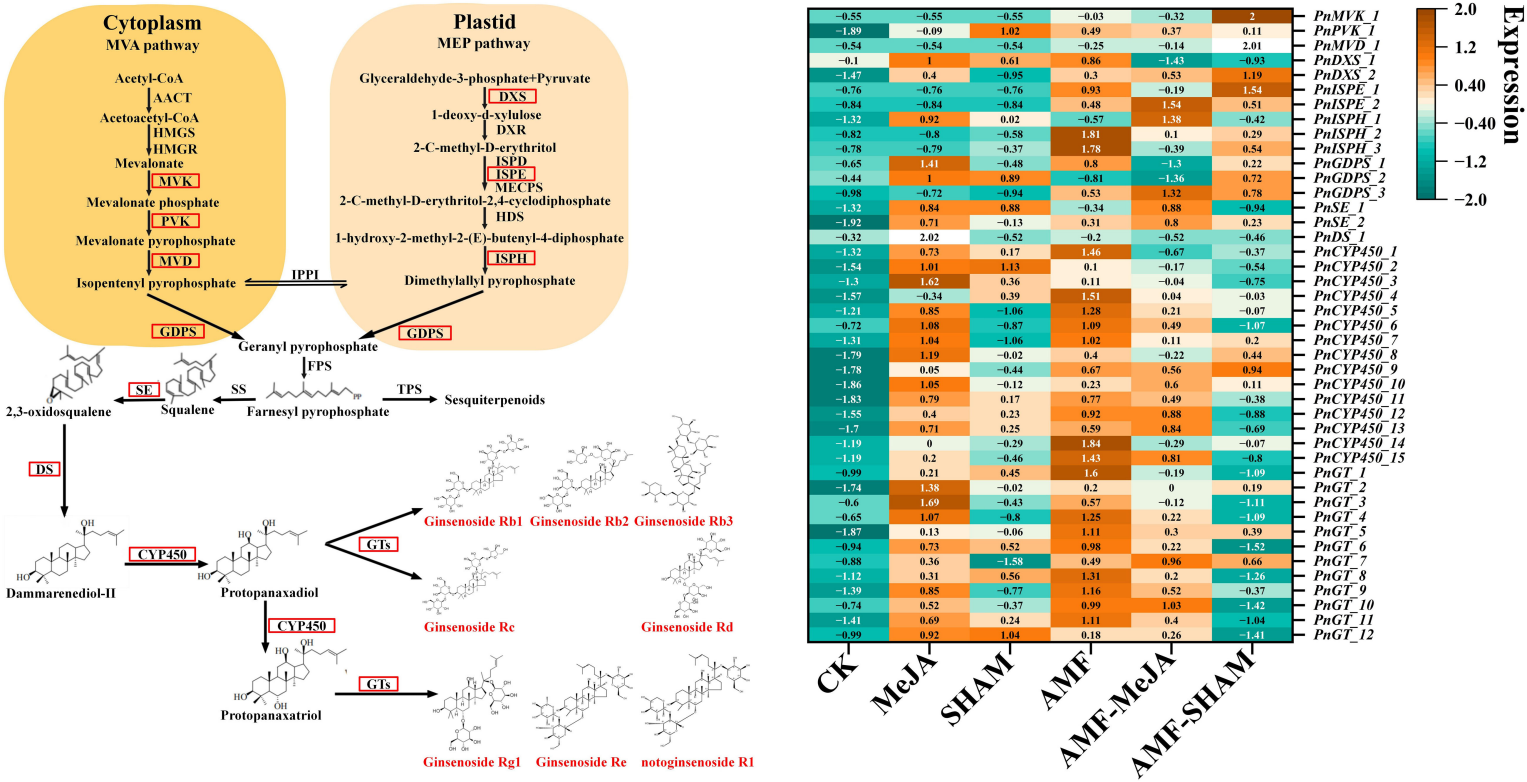
Expression profiles of transcripts encoding enzymes involved in notoginsenoside biosynthesis in *P. notoginseng* under AMF, MeJA and SHAM. The expression patterns are shown in a heatmap, which was constructed using the normalized FPKM value (Z). The normalized equation is $z=\frac{x-\bar{x}}{\sigma }$ , in which Z means the normalized value; χ means the average FPKM of three replicates in one group; $\bar{x}$ and σ mean the average FPKM and standard deviation of all groups, respectively. The color transition from bottle green to deep yellow indicates an increase in the expression level. The results showed that DEGs of CYP450 and GT may be largely responsible for the biosynthesis and accumulation of different types of notoginsenosides, especially in response to AMF colonization and MeJA addition. The expression levels of DEGs significantly increased under AMF inoculation and MeJA addition, e.g., *DXS*, *SE*, *CYP450* and *GT*. The expression fold changes of *PnISPH_2*, *PnISPH_3*, *PnCYP450_1*, *PnCYP450_4*, *PnCYP450_5*, *PnCYP450_6*, *PnCYP450_9*, *PnCYP450_12*, *PnCYP450_14*, *PnCYP450_15*, *PnGT_1*, *PnGT_4*, *PnGT_5*, *PnGT_6*, *PnGT_8*, *PnGT_9* and *PnGT_11* in the AMF group were larger than those in the MeJA and AMF-MeJA groups. Abbreviations: AACT, acetyl-CoA acetyltransferase; CYP450, cytochrome P450 monooxygenase; DMAPP, dimethylallyl diphosphate; DS, dammarenediol synthase; DXR, γ-deoxy-D-xylulose-5-phosphate reductoisomerase; DXS, 1-deoxy-D-xylulose-5-phosphate synthase; FPP, farnesyl pyrophosphate; GDPS, geranyl diphosphatesynthase; GT, glycosyltransferase; HDS, 4-hydroxy-3-methylbut-2-(*E*)-enyl diphosphate synthase; HMGR, 3-hydroxy-3-methyl-glutaryl-CoA reductase; HMGS, 3-hydroxy-3-methyl-glutaryl-CoA synthase; ISPD, 2-C-methyl-d-erythritol-4-phosphate cytidyltransferase; ISPE, diphosphocytidyl-2-C-methyl-d-erythritol kinase; ISPH, (E)-4-hydroxy-3-methylbut-2-enyl pyrophosphate reductase; MECPS, 2-C-methyl-D-erythritol-2, 4-cyclodiphosphate synthase; MEP, methylerythritol phosphate; MVA, mevalonate; MVD, diphosphomevalonate decarboxylase; MVK, mevalonate kinase; PVK, phophomevalonate diphosphate kinase; SE, squalene epoxidase; SS, squalene synthase; TPS, sesquiterpene synthases.

Notoginsenoside biosynthesis in roots is regulated by various environmental factors and directly results in change in the expression of key enzyme-encoding genes. In total, 381 DEGs were found in the six transcriptome libraries of AMF, MeJA, AMF-MeJA, SHAM, AMF-SHAM and CK, in which 1, 3, 1, 5, 1, 4, 4, 8, 7, 3, 186 and 147 unigenes were clustered into the families mevalonate kinase (*MVK*), phophomevalonate diphosphate kinase (*PVK*), diphosphomevalonate decarboxylase (*MVD*), 1-deoxy-D-xylulose-5-phosphate synthase (*DXS*), γ-deoxy-D-xylulose-5-phosphate reductoisomerase (*DXR*), diphosphocytidyl-2-C-methyl-d-erythritol kinase (*ISPE*), (E)-4-hydroxy-3-methylbut-2-enyl pyrophosphate reductase (*ISPH*), geranyl diphosphatesynthase (*GDPS*), *SE, DS*, *CYP450* and *GT*, respectively (**Supplementary Table 11**). The DEGs of CYP450 and GT may be largely responsible for the biosynthesis and accumulation of different types of notoginsenosides, especially in response to AMF colonization and MeJA addition. As shown in **Figure 6**, the expression levels of DEGs significantly increased under AMF inoculation and MeJA addition, e.g., *DXS*, *SE*, *CYP450* and *GT*. The expression fold changes of *PnISPH_2*, *PnISPH_3*, *PnCYP450_1*, *PnCYP450_4*, *PnCYP450_5*, *PnCYP450_6*, *PnCYP450_9*, *PnCYP450_12*, *PnCYP450_14*, *PnCYP450_15*, *PnGT_1*, *PnGT_4*, *PnGT_5*, *PnGT_6*, *PnGT_8*, *PnGT_9* and *PnGT_11* in the AMF group were larger than those in the MeJA and AMF-MeJA groups. Compared to AMF, the expression levels of most DEGs listed in the heatmap decreased significantly under AMF-SHAM, suggesting that the addition of SHAM can inhibit the activation of AMF in saponin biosynthesis. A large difference in the expression levels of DEGs, e.g., *PnDXS_2*, *PnISPH_1*, *PnGDPS_1*, *PnSE_2*, *PnDS_1*, *PnCYP450_5*, *PnCYP450_6*, *PnCYP450_7*, *PnCYP450_8*, *PnCYP450_10*, *PnGT_2*, *PnGT_3*, *PnGT_4*, *PnGT_9* and *PnGT_10*, among MeJA, SHAM and CK showed that JA played a positive promoting role, while SHAM had no obvious effect.

Key enzyme-encoding genes involved in JA biosynthesis responded to the treatments of AMF, MeJA and SHAM, and were differentially expressed (**Supplementary Table 12**). *PnLOX-1* and *PnLOX-3* significantly upregulated their expression levels in response to AMF, and the relative expression levels of *PnLOX-2* and *PnOPR-1* increased under MeJA and AMF-SHAM. In general, the effects of AMF, MeJA and AMF-SHAM on the expression of key enzyme-encoding genes were not exactly the same, thereafter affecting the endogenous concentrations of JAs.

### 3.8 Correlation analysis among saponin contents, key enzyme activities and DEGs

**Figure 7:**
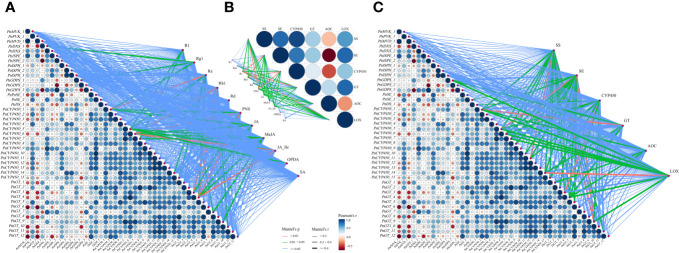
Correlation analysis among saponin contents, key enzyme activities and DEGs. **(A)** Correlations between DEGs and key enzyme activities involved in saponin and JA biosynthesis. **(B)** Correlations between key enzyme activities and saponin and JA contents. **(C)** Correlations between DEGs and saponin and JA contents. The results showed that a high correlation between any two of saponin contents, key enzyme activities and expression levels of DEGs. Edge width corresponds to the absolute value of the correlation coefficient determined by the linear mixed-effects models. Colors indicate correlation types. Red, green and blue lines denote highly significant (P < 0.01), significant (P = 0.01~0.05) and non-significant (P > 0.05) correlations, respectively, based on Mantel test. Pairwise comparisons of DEGs or enzyme activities are shown in the triangle, with a color gradient from red to blue (low to high) denoting Pearson’s correlation coefficient.

As shown in **Figure 7A**, there was a high correlation between expression levels of DEGs involved in notoginsenoside synthesis and activities of key enzymes that catalyzes the synthesis of saponins and JAs. Obviously, the contents of five monomeric saponins and PNSs and the concentrations of JAs were closely related to activities of key enzymes SS, SE, CYP450, GT, AOC and LOX (**Figure 7B**). In addition, expression levels of DEGs were also well correlated with contents of saponins and JAs (**Figure 7C).** These results fully showed that JAs mediated the synthesis of saponins and responded to the induction of AMF that representing by DEGs. Pearson’s correlation results showed there were generally high correlation among DEGs or key enzyme activities.

## 4 Discussion

As a widely used biological factor, AMF contribute to the growth and development of the host during its symbiosis with plants and have a positive effect on improving the production and accumulation of active compounds in medicinal plants. The biomass of some medicinal plants, e.g., *Salvia officinalis* L. and *Melissa officinalis* L., increased significantly after inoculation with the single agent *Septoglomus viscosum* and combined *Scutellospora heterogama*, *Claroideoglomus etunicatum* and *Acaulospora morrowiae* agents (Tarraf et al., 2017; de Assis et al., 2020). In addition, increasing evidence has indicated that the contents of many secondary metabolites, such as *Passiflora alata* Curtis saponin under *A. longula* (Muniz et al., 2021), *O. basilicum* essential oil under mixed AMF fertilizer containing *Rhizophagus irregularis*, *Funneliformis mosseae*, *G. aggregatum* (Yilmaz and Karik, 2022) and *Thymus vulgaris* terpenoids under mixed AMF inoculum of *R. intraradices* and *F. mosseae* (Arpanahi et al., 2020), increased in varying degrees by inoculating AMF on the roots. In this study, the *P. notoginseng* biomass and saponin contents significantly increased, including plant height, total fresh weight, fresh root weight, notoginsenoside R1, ginsenoside Rg1, Re, Rb1 and Rd, and PNSs under mixed AMF of *G. intraradices* and *G. etunicatum* (**Figure 1**). In fact, the promoting effect of AMF depends on the AMF species and their hosts (Diagne et al., 2020; Ran et al., 2021a; Amani Machiani et al., 2022). *R. irregularis*, *F. mosseae* and *C. etunicatum* are common AMF used to improve the contents of active substances and strengthen tolerance to stress (Ghanbarzadeh et al., 2019; Yilmaz and Karik, 2022; Tyagi et al., 2023).

Studies have shown that the accumulation of plant active substances is closely related to the activities and contents of key enzymes, of which the biosynthesis process is regulated by intrinsic mechanisms and external factors (Shah et al., 2022; He et al., 2023). Ran et al. (2021a) and Choi et al. (2005) found that the activities of key enzymes SS, SE and CYP450 in *P. quinquefolius* and *P. ginseng* significantly increased under the treatments of AMF and exogenous MeJA, which was similar to our studies that a significant increase in the activities of SS, SE, CYP450 and GT presented under the same treatments (**Figure 2**).

Evidence has shown that AMF participate in the biosynthesis of secondary metabolites either by directly improving the plant’s ability to obtain nutrients, water and minerals or indirectly stimulating secondary metabolite biosynthesis pathways by altering phytohormone concentrations and the production of signaling molecules, which in turn affects the production of secondary metabolites (Amani Machiani et al., 2022; Ran et al., 2022; Zhao et al., 2022). Currently, it is widely believed that phytohormones play a central role in the whole process of plant-microbiota interactions, which not only regulate plant growth and tolerance but also affect the diversity and versatility of plant microbial communities (Eichmann et al., 2021; Chen et al., 2023). Among the many plant hormones, JAs play a dominant role in regulating the synthesis of secondary metabolites and improving tolerance to stress, together with others (Yang et al., 2019), which can stimulate plant triterpenoid saponin accumulation by upregulating the expression of transcription factors and key genes (Mandal et al., 2015; Buraphaka and Putalun, 2020; Chen et al., 2023).

The results reported by Pedranzani et al. (2016) and Song et al. (2019) showed that the concentrations and activities of OPDA, JA, 12-OH-JA, JA-Ile and SA in *Digitaria eriantha* cv. and *Nicotiana attenuata* torr. Ex. Watson roots significantly increased under inoculation with *R. irregularis*, respectively. Mandal et al. (2015) also confirmed that the colonization of *R. intraradices* can elevate the JA concentration in *A. annua*, which was consistent with our findings that the levels of endogenous JAs, including JA, MeJA, JA-Ile and OPDA, increased significantly under AMF inoculation (**Figure 3**). The application of exogenous MeJA was developed as a noninvasive method to induce the accumulation of saponins and JAs. Many studies have shown that the addition of exogenous MeJA significantly increased the biomass and contents of triterpenoid saponins in *S. vaccaria* (Chen et al., 2023), *Leucas aspera* Spreng (Vijendra et al., 2020), *P. quinquefolium* (Kochan et al., 2018) and *O. basilicum* (Misra et al., 2017), which is in line with our results listed in **Figure 1**.

The addition of exogenous MeJA/JA greatly interferes with the biosynthesis and metabolism of MeJA/JA in plants, and it is generally believed that low concentrations of exogenous MeJA/JA have promoting effect, while high concentrations have inhibitory effect (Regvar et al., 1996; Ludwig-Müller et al., 2002; Formenti and Rasmann, 2019; Liu et al., 2022). The concentrations of JA, MeJA, JA-Ile and OPDA in our study increased significantly under the addition of MeJA (**Figure 3**). Similar results were confirmed by the reports of Yue et al. (2023) and Mandal et al. (2015) that β-citraurin and JA concentrations in *Citrus sinensis* cv. Newhall and *A. annua* increased under supplementation with MeJA. In addition, the activities of key enzymes AOC and LOX involved in JA biosynthesis improved greatly under AMF and MeJA, particularly in AMF (**Figure 4**). Similar results were also found in the study of Nair et al. (2015) in which inoculation of tomato with *G. fasiculatum* resulted in a significant elevation in enzyme LOX activity and MeJA levels, suggesting that AMF and MeJA were involved in the regulation of JA biosynthesis.

At present, there are few studies on the regulation of MeJA-mediated regulatory mechanisms of PNS accumulation under AMF inoculation, especially the dose-effect relationship among AMF colonization, endogenous JA concentration and notoginsenoside contents. In this study, the average colonization of AMF in fibrous roots was as high as 62.4%, which ensured that the roles of AMF were representative and persuasive. The interactive relationship among the three factors was initially elucidated under supplementation with 100 µmol/L MeJA. The above results revealed that notoginsenoside contents, JA concentrations and related key enzyme activities increased under AMF and MeJA, particularly in AMF. However, the effect of AMF largely weakened under the interactive treatment of AMF and MeJA, confirming the hypothesis that low concentrations improve and high concentrations inhibit. The addition of SHAM further verified the role of JA in the induction of AMF, as notoginsenoside contents and key enzyme activities presented nonsignificant differences between the CK and SHAM/AMF-SHAM groups. Unfortunately, the quantitative relationship among the AMF colonization rate, JA concentration and active substance content is still unclear, as is the regulatory pathway.

To reveal the mechanism of notoginsenoside accumulation mediated by MeJA under AMF inoculation, we not only focused on the changes in phytohormone concentrations and key enzyme activities but also analyzed the changes in genes involved in PNS biosynthesis in response to the induction of AMF, MeJA and SHAM using transcriptome sequencing, which has been used to obtain important transcriptome data of secondary metabolites and key enzyme sequences related to triterpenoid saponin biosynthesis from medicinal plants such as *P. ginseng* (Zhao et al., 2019; Li et al., 2021; Zhu et al., 2023) and *P. quinquefolium* (Peng et al., 2022). The DEGs in *P. notoginseng* roots were more susceptible to AMF colonization than the addition of MeJA and SHAM by pairwise comparisons of AMF vs. CK (3,553), MeJA vs. CK (3,405), SHAM vs. CK (3,522), AMF-MeJA vs. AMF (1,811), AMF-MeJA vs. MeJA (3,070), AMF-SHAM vs. AMF (800) and AMF-SHAM vs. SHAM (4,680). Similar results were found by Ran et al. (2023) and Ban et al. (2023) in *P. quinquefolius* and *Phragmites australis* (Cav.) Trin. ex Steud. under AMF. The DEGs involved in notoginsenoside biosynthesis were clustered into families *MVK*, *PVK*, MVD, *DXS*, *DXR*, *ISPE*, *ISPH*, GDPS, *SE*, DS, *CYP450* and *GT*, of which *CYP450* and *GT* were the two dominant families, with the number of 186 and 147, respectively (**Supplementary Table 11**). The key enzyme CYP450 mainly catalyzes the formation and diversification of functional groups of the triterpenoid skeleton such as hydroxyl oxidation, carbonyl groups and carboxyl groups (Cao et al., 2023a). The function of the key enzyme GT is mainly to further glycosylate the triterpenoids oxidatively modified by CYP450, thereby forming various plant triterpenoid glycosides or saponins (Rahimi et al., 2019; Li et al., 2022; Chen et al., 2023; Zhang et al., 2024).

The expression of many key enzyme-encoding genes, e.g., *PnISPH_1*, *PnDS_1*, *PnCYP450_5*, *PnCYP450_6*, *PnCYP450_7*, *PnCYP450_12*, *PnCYP450_15*, *PnGT_3*, *PnGT_4*, *PnGT_9* and *PnGT_10*, significantly increased under AMF and MeJA while most of these genes did not change significantly under SHAM or AMF-SHAM (**Figure 6**), directly supporting the results of significantly elevated notoginsenoside contents in the AMF and MeJA groups. However, the superposition treatment of AMF and MeJA did not increase the expression levels of these genes. This result was also consistent with the indices of biomass and saponin contents. Yuan et al. (2019) found that the genes related to metabolism involved in terpene skeleton biosynthesis in *Atractylodes lancea* (Thunb.) DC. were upregulated, and the contents of terpene compounds significantly increased under inoculation with the endophytic fungus *Gilmaniella* sp. AL12. The triterpene saponin contents in *Centella asiatica* (L.) Urban and the expression levels of key enzyme-encoding genes involved in triterpene biosynthesis also significantly increased in response to the application of exogenous MeJA (Yuan et al., 2019; Buraphaka and Putalun, 2020). The application of AMF agents or short-term treatment with MeJA is a preferred strategy to increase saponin accumulation in *P. notoginseng* cultivation or biosynthesis in a reactor.

The differential expression of key enzyme-encoding genes involved in JA showed that AMF, MeJA and SHAM affected the biosynthesis of JA and directly correlated with saponin accumulation. The DEGs that encode proteins catalyzing the formation of precursors for JA mainly clustered into families *LOX* and *OPR*, e.g., *PnLOX-1*, *PnLOX-2*, *PnLOX-3*, *PnOPR-1* and *PnOPR-2* (Wang Q. et al., 2021). Increasing evidence has shown that JA synthesis-related genes, e.g., *DXS*, *DXR* and *OPR3* in *P. quinquefolius* and *Solanum tuberosum* L. upregulate their expression levels in response to the induction of *R. intraradices* and *R. irregularis* (Schoenherr et al., 2019; Ran et al., 2021b and 2023). Wei et al. (2022) confirmed that in *Curcuma wenyujin* Y. Chen & C. Ling, the expression levels of key enzyme-encoding genes (*LOX*, *AOS*, *AOC*, *OPR*, *MFP* and *JAR*) involved in JA biosynthesis and JA signal transduction genes (*MYC2* and *JAZ*) increased significantly under MeJA. Similar results were found in our study: some genes, e.g., *PnLOX-1* and *PnLOX-3*, significantly upregulated their expression levels in response to AMF induction, and the relative expression levels of *PnLOX-2* and *PnOPR-1* increased under MeJA (**Supplementary Table 12**). Importantly, a high correlation among saponin contents, key enzyme activities and expression levels of DEGs (**Figure 7**) fully showed that JAs were involved in regulating the synthesis of saponins.

## 5 Conclusion

This study provides a novel perspective to reveal the role mechanism of AMF in improving the biosynthesis and accumulation of PNSs, in which the changes in JA concentrations and the activities of related key enzymes and genes involved in JA biosynthesis were systematically analyzed. The inoculation of AMF and addition of MeJA increased the biomass and saponin contents of *P. notoginseng*, the concentrations of JAs and the key enzyme activities involved in notoginsenoside biosynthesis, but the interactive treatment of AMF and MeJA weakened the effect of AMF, suggesting that a high concentration of endogenous JA may have inhibitory effect. In addition, the activities of key enzymes AOC and LOX involved in JA biosynthesis increased significantly, as did the concentrations of JA, MeJA, JA-Ile and OPDA, under AMF. Transcriptome sequencing results indicated that DEGs involved in notoginsenoside and JA biosynthesis were significantly enriched in response to AMF induction, e.g., upregulated genes of *PVKs*, *DXSs*, *ISPEs*, *ISPHs*, *GDPSs*, *SEs*, *CYP450s*, and *GTs*, while treatments AMF-MeJA and SHAM decreased the abundance and levels. Interestingly, a high correlation between any two of saponin contents, key enzyme activities and expression levels of DEGs. Taken together, it is confirmed that the inoculation of AMF can promote the growth and saponin accumulation of *P. notoginseng* by strengthening the activities of key enzymes and the expression levels of encoding genes, in which the JA regulatory pathway was a key link. This study provides references for implementing ecological planting of *P. notoginseng*, improving saponin accumulation and illustrating biosynthesis mechanisms.

## Data availability statement

The datasets presented in this study can be found in online repositories. The names of the repository/repositories and accession number(s) can be found in the article/**Supplementary Material**.

## Author contributions

H-YD: Methodology, Formal analysis, Writing – original draft. X-KZ: Writing – review & editing, Funding acquisition, Writing – original draft, Methodology, Formal analysis. YB: Writing – original draft, Methodology. DC: Writing – original draft, Methodology. X-NL: Writing – original draft, Methodology. YW: Writing – original draft, Methodology. G-HC: Writing – review & editing, Funding acquisition, Conceptualization, Writing – original draft. SH: Writing – review & editing, Writing – original draft, Supervision, Project administration, Funding acquisition, Conceptualization.

## Supplementary material

Supplementary Figure 1 Analysis of sample correlation by clustering heat map analysis. Treatment groups of AMF, MeJA, SHAM, AMF-MeJA and AMF-SHAM had strong correlation (R^2^ ≧ 0.75) between two duplicate samples. However, the correlation between CK and other groups was relative low with a range of 0.53~0.88.

Supplementary Figure 2 KEGG function classification of the differentially expressed genes of *P. notoginseng* fibrous root transcriptome under the treatments of AMF, MeJA and SHAM. A total of 91,991 unigenes were categorized into 25 functional KEGG clusters, 3,050 unigenes involved in saponin biosynthesis were classified as “secondary metabolites biosynthesis, transport and catabolism”, accounting for 1.92%.

Supplementary Table 8 GO classification of unigenes.

Supplementary Table 9 KEGG pathway of unigenes.

Supplementary Table 10 KOG classification of unigenes.

Supplementary Table 11 FPKM of DEGs involved in notoginsenoside biosynthesis.

Supplementary Table 12 FPKM of DEGs involved in JA biosynthesis.

Supplementary Table 13 Original data used for Mantel tests.

## References

[B1] Amani Machiani M. Javanmard A. Habibi Machiani R. Sadeghpour A. (2022). Arbuscular mycorrhizal fungi and changes in primary and secondary metabolites. Plants 11, 2183. doi: 10.3390/plants11172183 36079565 PMC9460575

[B2] Arpanahi A. A. Feizian M. Mehdipourian G. Khojasteh D. N. (2020). Arbuscular mycorrhizal fungi inoculation improve essential oil and physiological parameters and nutritional values of *Thymus daenensis* Celak and *Thymus vulgaris* L. under normal and drought stress conditions. Eur. J. Soil Biol. 100, 103217. doi: 10.1016/j.ejsobi.2020.103217

[B3] Bai Y. Liu H. Pan J. Zhang S. Guo Y. Xian Y.. (2021). Transcriptomics and metabolomics changes triggered by inflorescence removal in *Panax notoginseng* (Burk.). Front. Plant Sci. 12, 761821. doi: 10.3389/fpls.2021.761821 34868157 PMC8636121

[B4] Ban Y. Tan J. Xiong Y. Mo X. Jiang Y. Xu Z. (2023). Transcriptome analysis reveals the molecular mechanisms of *Phragmites australis* tolerance to CuO-nanoparticles and/or flood stress induced by arbuscular mycorrhizal fungi. J. Hazard. Mater. 442, 130118. doi: 10.1016/j.jhazmat.2022.130118 36303351

[B5] Buchfink B. Xie C. Huson D. H. (2015). Fast and sensitive protein alignment using DIAMOND. Nat. Methods 12, 59–60. doi: 10.1038/nmeth.3176 25402007

[B6] Buraphaka H. Putalun W. (2020). Stimulation of health-promoting triterpenoids accumulation in *Centella asiatica* (L.) Urban leaves triggered by postharvest application of methyl jasmonate and salicylic acid elicitors. Ind. Crops Prod. 146, 112171. doi: 10.1016/j.indcrop.2020.112171

[B7] Cai B. D. Zhu J. X. Gao Q. Luo D. Yuan B. F. Feng Y. Q. (2014). Rapid and high-throughput determination of endogenous cytokinins in *Oryza sativa* by bare Fe3O4 nanoparticles-based magnetic solid-phase extraction. J. Chromatogr. A 1340, 146–150. doi: 10.1016/j.chroma.2014.03.030 24685168

[B8] Camargo-Ricalde S. L. (2002). Dispersal, distribution and establishment of arbuscular mycorrhizal fungi: a review. Bot. Sci. 71, 33–34. doi: 10.17129/botsci.1661

[B9] Cao G. H. Bai X. Zhang C. R. Li X. G. Dai H. Y. Bi Y.. (2023a). Physiological response and transcriptome profiling reveal phosphate-mediated amelioration of arsenic accumulation and toxicity in *Panax notoginseng.* Environ. Exp. Bot. 206, 105136. doi: 10.1016/j.envexpbot.2022.105136

[B10] Cao G. H. Li X. G. Zhang C. R. Xiong Y. R. Li X. Li T.. (2023b). Physiological response mechanism of heavy metal-resistant endophytic fungi isolated from the roots of *Polygonatum kingianum.* Env. Microbiol. Rep. 15, 568–581. doi: 10.1111/1758-2229.13194 PMC10667662 37604512

[B11] Chen S. Zhou Y. Chen Y. Gu J. (2018). fastp: an ultra-fast all-in-one FASTQ preprocessor. Bioinformatics 34, i884–i890. doi: 10.1093/bioinformatics/bty560 30423086 PMC6129281

[B12] Chen X. Hudson G. A. Mineo C. Amer B. Baidoo E. E. Crowe S. A.. (2023). Deciphering triterpenoid saponin biosynthesis by leveraging transcriptome response to methyl jasmonate elicitation in *Saponaria vaccaria.* Nat. Commun. 14, 7101. doi: 10.1038/s41467-023-42877-0 PMC10625584 37925486

[B13] Cheng J. Chen J. Liao J. Wang T. Shao X. Long J.. (2023). High-throughput transcriptional profiling of perturbations by *Panax ginseng* saponins and *Panax notoginseng* saponins using TCM-seq. J. Pharm. Anal. 13, 376–387. doi: 10.1016/j.jpha.2023.02.009 37181291 PMC10173292

[B14] Cheng Y. Liu H. Tong X. Liu Z. Zhang X. Li D.. (2020). Identification and analysis of CYP450 and UGT supergene family members from the transcriptome of *Aralia elata* (Miq.) seem reveal candidate genes for triterpenoid saponin biosynthesis. BMC Plant Biol. 20, 214. doi: 10.1186/s12870-020-02411-6 32404131 PMC7218531

[B15] Chini A. Monte I. Zamarreño A. M. Hamberg M. Lassueur S. Reymond P.. (2018). An OPR3-independent pathway uses 4, 5-didehydrojasmonate for jasmonate synthesis. Nat. Chem. Biol. 14, 171–178. doi: 10.1038/nchembio.2540 29291349

[B16] Choi D. W. Jung J. Ha Y. I. Park H. W. In D. S. Chung H. J.. (2005). Analysis of transcripts in methyl jasmonate-treated ginseng hairy roots to identify genes involved in the biosynthesis of ginsenosides and other secondary metabolites. Plant Cell Rep. 23, 557–566. doi: 10.1007/s00299-004-0845-4 15538577

[B17] Cui K. Lin Y. Zhou X. Li S. Liu H. Zeng F.. (2015). Comparison of sample pretreatment methods for the determination of multiple phytohormones in plant samples by liquid chromatography–electrospray ionization-tandem mass spectrometry. Microchem. J. 121, 25–31. doi: 10.1016/j.microc.2015.02.004

[B18] Cun Z. Zhang J. Y. Hong J. Yang J. Gao L. L. Hao B.. (2024). Integrated metabolome and transcriptome analysis reveals the regulatory mechanism of low nitrogen-driven biosynthesis of saponins and flavonoids in *Panax notoginseng* . Gene. 901, 148163. doi: 10.1016/j.gene.2024.148163 38224922

[B19] de Assis R. M. A. Carneiro J. J. Medeiros A. P. R. de Carvalho A. A. da Cunha Honorato A. Carneiro M. A. C.. (2020). Arbuscular mycorrhizal fungi and organic manure enhance growth and accumulation of citral, total phenols, and flavonoids in *Melissa officinalis* L. Ind. Crops Prod. 158, 112981. doi: 10.1016/j.indcrop.2020.112981

[B20] Diagne N. Ngom M. Djighaly P. I. Fall D. Hocher V. Svistoonoff S. (2020). Roles of arbuscular mycorrhizal fungi on plant growth and performance: Importance in biotic and abiotic stressed regulation. Diversity 12, 370. doi: 10.3390/d12100370

[B21] Dinday S. Ghosh S. (2023). Recent advances in triterpenoid pathway elucidation and engineering. Biotechnol. Adv. 68, 108214. doi: 10.1016/j.biotechadv.2023.108214 37478981

[B22] Domokos E. Jakab-Farkas L. Darkó B. Bíró-Janka B. Mara G. Albert C.. (2018). Increase in *Artemisia annua* plant biomass artemisinin content and guaiacol peroxidase activity using the arbuscular mycorrhizal fungus *Rhizophagus irregularis* . Front. Plant Sci. 9, 478. doi: 10.3389/fpls.2018.00478 29706981 PMC5908966

[B23] Eichmann R. Richards L. Schäfer P. (2021). Hormones as go-betweens in plant microbiome assembly. Plant J. 105, 518–541. doi: 10.1111/tpj.15135 33332645 PMC8629125

[B24] Faizal A. Sari A. V. (2019). Enhancement of saponin accumulation in adventitious root culture of Javanese ginseng (*Talinum paniculatum* Gaertn.) through methyl jasmonate and salicylic acid elicitation. Afr. J. Biotechnol. 18, 130–135. doi: 10.5897/AJB

[B25] Fan D. Ji M. Wu J. Chen H. Jia H. Zhang X.. (2023). Grazing does not influence soil arbuscular mycorrhizal fungal diversity, but increases their interaction complexity with plants in dry grasslands on the Tibetan Plateau. Ecol. Indic. 148, 110065. doi: 10.1016/j.ecolind.2023.110065

[B26] Floková K. Tarkowská D. Miersch O. Strnad M. Wasternack C. Novák O. (2014). UHPLC–MS/MS based target profiling of stress-induced phytohormones. Phytochemistry 105, 147–157. doi: 10.1016/j.phytochem.2014.05.015 24947339

[B27] Foo E. Ross J. J. Jones W. T. Reid J. B. (2013). Plant hormones in arbuscular mycorrhizal symbioses: an emerging role for gibberellins. Ann. Bot.-London 111, 769–779. doi: 10.1093/aob/mct041 PMC3631329 23508650

[B28] Formenti L. Rasmann S. (2019). Mycorrhizal fungi enhance resistance to herbivores in tomato plants with reduced jasmonic acid Production. Agronomy 9, 131. doi: 10.3390/agronomy9030131

[B29] Ghanbarzadeh Z. Mohsenzadeh S. Rowshan V. Moradshahi A. (2019). Evaluation of the growth, essential oil composition and antioxidant activity of *Dracocephalum moldavica* under water deficit stress and symbiosis with *Claroideoglomus etunicatum* and *Micrococcus yunnanensis* . Sci. Hortic. 256, 108652. doi: 10.1016/j.scienta.2019.108652

[B30] Hause B. Mrosk C. Isayenkov S. Strack D. (2007). Jasmonates in arbuscular mycorrhizal interactions. Phytochemistry 68, 101–110. doi: 10.1016/j.phytochem.2006.09.025 17097695

[B31] He J. Yao L. Pecoraro L. Liu C. Wang J. Huang L.. (2023). Cold stress regulates accumulation of flavonoids and terpenoids in plants by phytohormone, transcription process, functional enzyme, and epigenetics. Crit. Rev. Biotechnol. 43, 680–697. doi: 10.1080/07388551.2022.2053056 35848841

[B32] Hu W. Zheng Y. Zheng J. Yan K. Liang Z. Xia P. (2022). Binding proteins PnCOX11 and PnDCD strongly respond to GA and ABA in *Panax notoginseng.* Int. J. Biol. Macromol. 212, 303–313. doi: 10.1016/j.ijbiomac.2022.05.134 35609837

[B33] Huang H. (2021) linkET: Everything is Linkable. R package version 0.0.3. Available online at: https://github.com/Hy4m/linKET.

[B34] Huang C. Li P. Niu T. Zhao S. Yang L. Wang R.. (2023). Integrated transcriptome and proteome analyses reveal candidate genes for ginsenoside biosynthesis in *Panax japonicus* C. A. Meyer. Front. Plant Sci. 13, 1106145. doi: 10.3389/fpls.2022.1106145 36699857 PMC9868605

[B35] Jiang Z. Tu L. Yang W. Zhang Y. Hu T. Ma B.. (2021). The chromosome-level reference genome assembly for *Panax notoginseng* and insights into ginsenoside biosynthesis. Plant Commun. 2, 100113. doi: 10.1016/j.xplc.2020.100113 33511345 PMC7816079

[B36] Kazan K. (2015). Diverse roles of jasmonates and ethylene in abiotic stress tolerance. Trends Plant Sci. 20, 219–229. doi: 10.1016/j.tplants.2015.02.001 25731753

[B37] Kochan E. Balcerczak E. Lipert A. Szymańska G. Szymczyk P. (2018). Methyl jasmonate as a control factor of the synthase squalene gene promoter and ginsenoside production in American ginseng hairy root cultured in shake flasks and a nutrient sprinkle bioreactor. Ind. Crops Prod. 115, 182–193. doi: 10.1016/j.indcrop.2018.02.036

[B38] Kumar S. Arora N. Upadhyay H. (2021). “Arbuscular mycorrhizal fungi: Source of secondary metabolite production in medicinal plants,” in New and future developments in microbial biotechnology and bioengineering (Elsevier, Netherlands), 155–164. doi: 10.1016/B978-0-12-821005-5.00011-9

[B39] Langmead B. Salzberg S. L. (2012). Fast gapped-read alignment with Bowtie 2. Nat. Methods 9, 357–359. doi: 10.1038/nmeth.1923 22388286 PMC3322381

[B40] Lawrence M. Huber W. Pagès H. Aboyoun P. Carlson M. Gentleman R.. (2013). Software for computing and annotating genomic ranges. PloS Comput. Biol. 9, e1003118. doi: 10.1371/journal.pcbi.1003118 23950696 PMC3738458

[B41] Li B. Dewey C. N. (2011). RSEM: accurate transcript quantification from RNA-Seq data with or without a reference genome. BMC Bioinf. 12, 1–16. doi: 10.1186/1471-2105-12-323 PMC3163565 21816040

[B42] Li Y. Li J. Diao M. Peng L. Huang S. Xie N. (2022). Characterization of a group of UDP-glycosyltransferases involved in the biosynthesis of triterpenoid saponins of *Panax notoginseng.* ACS Synth. Biol. 11, 770–779. doi: 10.1021/acssynbio.1c00469 35107265

[B43] Li W. Li W. Yang S. Ma Z. Zhou Q. Mao J.. (2020). Transcriptome and metabolite conjoint analysis reveals that exogenous methyl jasmonate regulates monoterpene synthesis in grape berry skin. J. Agric. Food Chem. 68, 5270–5281. doi: 10.1021/acs.jafc.0c00476 32338508

[B44] Li M. Ma M. Wu Z. Liang X. Zheng Q. Li D.. (2023). Advances in the biosynthesis and metabolic engineering of rare ginsenosides. Appl. Microbiol. Biot. 107, 1–14. doi: 10.1007/s00253-023-12549-6 37126085

[B45] Li L. Wang Y. Zhao M. Wang K. Sun C. Zhu L.. (2021). Integrative transcriptome analysis identifies new oxidosqualene cyclase genes involved in ginsenoside biosynthesis in Jilin ginseng. Genomics 113, 2304–2316. doi: 10.1016/j.ygeno.2021.05.023 34048908

[B46] Li Y. Zhou C. Yan X. Zhang J. Xu J. (2016). Simultaneous analysis of ten phytohormones in *Sargassum horneri* by high-performance liquid chromatography with electrospray ionization tandem mass spectrometry. J. Sep. Sci. 39, 1804–1813. doi: 10.1002/jssc.201501239 26990813

[B47] Lin J. Wang Y. Sun S. Mu C. Yan X. (2017). Effects of arbuscular mycorrhizal fungi on the growth, photosynthesis and photosynthetic pigments of *Leymus chinensis* seedlings under salt-alkali stress and nitrogen deposition. Sci. Total Environ. 576, 234–241. doi: 10.1016/j.scitotenv.2016.10.091 27788438

[B48] Liu H. Lu X. Hu Y. Fan X. (2020). Chemical constituents of *Panax ginseng* and *Panax notoginseng* explain why they differ in therapeutic efficacy. Pharmacol. Res. 161, 105263. doi: 10.1016/j.phrs.2020.105263 33127555

[B49] Liu H. Tang H. Ni X. Zhang Y. Wang Y. (2022). Impact of an arbuscular mycorrhizal fungal inoculum and exogenous methyl jasmonate on the performance of tall fescue under saline-alkali condition. Front. Microbiol. 13, 902667. doi: 10.3389/fmicb.2022.902667 36160269 PMC9493314

[B50] Love M. I. Huber W. Anders S. (2014). Moderated estimation of fold change and dispersion for RNA-seq data with DESeq2. Genome Biol. 15, 1–21. doi: 10.1186/s13059-014-0550-8 PMC4302049 25516281

[B51] Ludwig-Müller J. (2010). “Hormonal responses in host plants triggered by arbuscular mycorrhizal fungi,” in Arbuscular mycorrhizas: physiology and function. Eds. Koltai H. Kapulnik Y. (Springer, Dordrecht), 169–190. doi: 10.1007/978-90-481-9489-6_8

[B52] Ludwig-Müller J. Bennett R. N. García-Garrido J. M. Piché Y. Vierheilig H. (2002). Reduced arbuscular mycorrhizal root colonization in *Tropaeolum majus* and *Carica papaya* after jasmonic acid application can not be attributed to increased glucosinolate levels. J. Plant Physiol. 159, 517–523. doi: 10.1078/0176-1617-00731

[B53] Majewska M. L. Rola K. Zubek S. (2017). The growth and phosphorus acquisition of invasive plants *Rudbeckia laciniata* and *Solidago gigantea* are enhanced by arbuscular mycorrhizal fungi. Mycorrhiza 27, 83–94. doi: 10.1007/s00572-016-0729-9 27581153 PMC5237450

[B54] Malar C. Wang Y. Stajich J. E. Kokkoris V. Villeneuve-Laroche M. Yildirir G.. (2022). Early branching arbuscular mycorrhizal fungus *Paraglomus occultum* carries a small and repeat-poor genome compared to relatives in the Glomeromycotina. Microb. Genomics 8, 000810. doi: 10.1099/mgen.0.000810 PMC9453076 35451944

[B55] Mandal S. Upadhyay S. Wajid S. Ram M. Jain D. C. Singh V. P.. (2015). Arbuscular mycorrhiza increase artemisinin accumulation in *Artemisia annua* by higher expression of key biosynthesis genes via enhanced jasmonic acid levels. Mycorrhiza 25, 345–357. doi: 10.1007/s00572-014-0614-3 25366131

[B56] Marro N. Grilli G. Soteras F. Caccia M. Longo S. Cofré N.. (2022). The effects of arbuscular mycorrhizal fungal species and taxonomic groups on stressed and unstressed plants: a global meta-analysis. New Phytol. 235, 320–332. doi: 10.1111/nph.18102 35302658

[B57] Misra R. C. Sharma S. Sandeep Garg A. Chanotiya C. S. Ghosh S. (2017). Two CYP716A subfamily cytochrome P450 monooxygenases of sweet basil play similar but nonredundant roles in ursane-and oleanane-type pentacyclic triterpene biosynthesis. New Phytol. 214, 706–720. doi: 10.1111/nph.14412 28967669

[B58] Muniz B. C. Falcão E. L. de Paula Monteiro R. dos Santos E. L. Bastos Filho C. J. A. da Silva F. S. B. (2021). *Acaulospora longula* Spain & NC Schenck: A low-cost bioinsumption to optimize phenolics and saponins production in *Passiflora alata* Curtis. Ind. Crops Prod. 167, 113498. doi: 10.1016/j.indcrop.2021.113498

[B59] Nair A. Kolet S. P. Thulasiram H. V. Bhargava S. (2015). Role of methyl jasmonate in the expression of mycorrhizal induced resistance against *Fusarium oxysporum* in tomato plants. Physiol. Mol. Plant P. 92, 139–145. doi: 10.1016/j.pmpp.2015.10.002

[B60] Ning Y. Ding Y. K. Chang Y. H. Zhang S. An H. M. Fu Y. J. (2023). Low concentration of MeJA-elicited the positive growth of *Rosa Roxburghii* via balancing phytohormone signal transduction and triterpenoids synthesis. Plant Growth Regul. 101, 187–199. doi: 10.1007/s10725-023-01012-1

[B61] Niu Q. Zong Y. Qian M. Yang F. Teng Y. (2014). Simultaneous quantitative determination of major plant hormones in pear flowers and fruit by UPLC/ESI-MS/MS. Anal. Methods 6, 1766–1773. doi: 10.1039/C3AY41885E

[B62] Pan J. Huang C. Yao W. Niu T. Yang X. Wang R. (2023). Full-length transcriptome, proteomics and metabolite analysis reveal candidate genes involved triterpenoid saponin biosynthesis in *Dipsacus asperoides.* Front. Plant Sci. 14, 1134352. doi: 10.3389/fpls.2023.1134352 PMC9950739 36844092

[B63] Pan X. Welti R. Wang X. (2010). Quantitative analysis of major plant hormones in crude plant extracts by high-performance liquid chromatography–mass spectrometry. Nat. Protoc. 5, 986–992. doi: 10.1038/nprot.2010.37 20448544

[B64] Pedranzani H. Rodríguez-Rivera M. Gutiérrez M. Porcel R. Hause B. Ruiz-Lozano J. M. (2016). Arbuscular mycorrhizal symbiosis regulates physiology and performance of *Digitaria eriantha* plants subjected to abiotic stresses by modulating antioxidant and jasmonate levels. Mycorrhiza 26, 141–152. doi: 10.1007/s00572-015-0653-4 26184604

[B65] Peng D. I. Yan Y. A. N. Ping W. A. N. G. Min Y. A. N. Ying-Ping W. A. N. G. Huang L. Q. (2022). Integrative SMRT sequencing and ginsenoside profiling analysis provide insights into the biosynthesis of ginsenoside in *Panax quinquefolium.* Chin. J. Nat. Med. 20, 614–626. doi: 10.1016/S1875-5364(22)60198-5 36031233

[B66] Qiu X. Xu Y. Xiong B. Dai L. Huang S. Dong T.. (2020). Effects of exogenous methyl jasmonate on the synthesis of endogenous jasmonates and the regulation of photosynthesis in citrus. Physiol. Plantarum 170, 398–414. doi: 10.1111/ppl.13170 32691420

[B67] Rahimi S. Kim J. Mijakovic I. Jung K. H. Choi G. Kim S. C.. (2019). Triterpenoid-biosynthetic UDP-glycosyltransferases from plants. Biotechnol. Adv. 37, 107394. doi: 10.1016/j.biotechadv.2019.04.016 31078628

[B68] Ran Z. Chen X. Li R. Duan W. Zhang Y. Fang L.. (2023). Transcriptomics and metabolomics reveal the changes induced by arbuscular mycorrhizal fungi in *Panax quinquefolius* L. J. Sci. Food Agric. 103, 4919–4933. doi: 10.1002/jsfa.12563 36942522

[B69] Ran Z. Ding W. Cao S. Fang L. Zhou J. Zhang Y. (2022). Arbuscular mycorrhizal fungi: Effects on secondary metabolite accumulation of traditional Chinese medicines. Plant Biol. 24, 932–938. doi: 10.1111/plb.13449 35733285

[B70] Ran Z. Yang X. Zhang Y. Zhou J. Guo L. (2021a). Effects of arbuscular mycorrhizal fungi on photosynthesis and biosynthesis of ginsenoside in *Panax quinquefolius* L. Theor. Exp. Plant Physiol. 33, 235–248. doi: 10.1007/s40626-021-00208-y

[B71] Ran Z. Yang X. Zhang Y. Zhou J. Guo L. (2021b). Transcriptional responses for biosynthesis of ginsenoside in arbuscular mycorrhizal fungi-treated *Panax quinquefolius* L. seedlings using RNA-seq. Plant Growth Regul. 95, 83–96. doi: 10.1007/s10725-021-00727-3

[B72] Regvar M. Gogala N. Zalar P. (1996). Effects of jasmonic acid on mycorrhizal *Allium sativum* . New Phytol. 134, 703–707. doi: 10.1111/j.1469-8137.1996.tb04936.x 33863203

[B73] Ren Y. Che X. Liang J. Wang S. Han L. Liu Z.. (2021). Brassinosteroids benefit plants performance by augmenting arbuscular mycorrhizal symbiosis. Microbiol. Spectr. 9, e01645–e01621. doi: 10.1128/spectrum.01645-21 34908500 PMC8672874

[B74] Robinson M. D. McCarthy D. J. Smyth G. K. (2010). edgeR: a Bioconductor package for differential expression analysis of digital gene expression data. Bioinformatics 26, 139–140. doi: 10.1093/bioinformatics/btp616 19910308 PMC2796818

[B75] Schoenherr A. P. Rizzo E. Jackson N. Manosalva P. Gomez S. K. (2019). Mycorrhiza-induced resistance in potato involves priming of defense responses against cabbage looper (Noctuidae: Lepidoptera). Environ. Entomol. 48, 370–381. doi: 10.1093/ee/nvy195 30715218

[B76] Shah S. Li X. Jiang Z. Fahad S. Hassan S. (2022). Exploration of the phytohormone regulation of energy storage compound accumulation in microalgae. Food Energy Secur. 11, e418. doi: 10.1002/fes3.418

[B77] Sharma A. Rather G. A. Misra P. Dhar M. K. Lattoo S. K. (2019). Jasmonate responsive transcription factor WsMYC2 regulates the biosynthesis of triterpenoid withanolides and phytosterol via key pathway genes in *Withania somnifera* (L.) Dunal. Plant Mol. Biol. 100, 543–560. doi: 10.1007/s11103-019-00880-4 31090025

[B78] Shen W. Song Z. Zhong X. Huang M. Shen D. Gao P.. (2022). Sangerbox: A comprehensive, interaction-friendly clinical bioinformatics analysis platform. iMeta 1, e36. doi: 10.1002/imt2.36 PMC10989974 38868713

[B79] Šimura J. Antoniadi I. Široká J. Tarkowská D. E. Strnad M. Ljung K.. (2018). Plant hormonomics: multiple phytohormone profiling by targeted metabolomics. Plant Physiol. 177, 476–489. doi: 10.1104/pp.18.00293 29703867 PMC6001343

[B80] Song Y. Wang M. Zeng R. Groten K. Baldwin I. T. (2019). Priming and filtering of antiherbivore defences among *Nicotiana attenuata* plants connected by mycorrhizal networks. Plant Cell Environ. 42, 2945–2961. doi: 10.1111/pce.13626 31348534

[B81] Sun R. T. Zhang Z. Z. Liu M. Y. Feng X. C. Zhou N. Feng H. D.. (2022). Arbuscular mycorrhizal fungi and phosphorus supply accelerate main medicinal component production of *Polygonum cuspidatum* . Front. Microbiol. 13, 1006140. doi: 10.3389/fmicb.2022.1006140 36160193 PMC9493279

[B82] Tamura K. Teranishi Y. Ueda S. Suzuki H. Kawano N. Yoshimatsu K.. (2017). Cytochrome P450 monooxygenase CYP716A141 is a unique β-amyrin C-16β oxidase involved in triterpenoid saponin biosynthesis in *Platycodon grandiflorus* . Plant Cell Physiol. 58, 874–884. doi: 10.1093/pcp/pcx043 28371833

[B83] Tarraf W. Ruta C. Tagarelli A. De Cillis F. De Mastro G. (2017). Influence of arbuscular mycorrhizae on plant growth, essential oil production and phosphorus uptake of *Salvia officinalis* L. Ind. Crops Prod. 102, 144–153. doi: 10.1016/j.indcrop.2017.03.010

[B84] Tyagi J. Mishra A. Kumari S. Singh S. Agarwal H. Pudake R. N.. (2023). Deploying a microbial consortium of Serendipita indica, *Rhizophagus intraradices*, and *Azotobacter chroococcum* to boost drought tolerance in maize. Environ. Exp. Bot. 206, 105142. doi: 10.1016/j.envexpbot.2022.105142

[B85] Varet H. Brillet-Guéguen L. Coppée J. Y. Dillies M. A. (2016). SARTools: a DESeq2-and EdgeR-based R pipeline for comprehensive differential analysis of RNA-Seq data. PloS One 11, e0157022. doi: 10.1371/journal.pone.0157022 27280887 PMC4900645

[B86] Vijendra P. D. Jayanna S. G. Kumar V. Sannabommaji T. Rajashekar J. Gajula H. (2020). Product enhancement of triterpenoid saponins in cell suspension cultures of *Leucas aspera* Spreng. Ind. Crops Prod. 156, 112857. doi: 10.1016/j.indcrop.2020.112857

[B87] Viswanath K. K. Varakumar P. Pamuru R. R. Basha S. J. Mehta S. Rao A. D. (2020). Plant lipoxygenases and their role in plant physiology. J. Plant Biol. 63, 83–95. doi: 10.1007/s12374-020-09241-x

[B88] Wang Q. Chen B. Chen X. Mao X. Fu X. (2023). Squalene epoxidase (SE) gene related to triterpenoid biosynthesis assists to select elite genotypes in medicinal plant: *Cyclocarya paliurus* (Batal.) Iljinskaja. Plant Physiol. Bioch. 199, 107726. doi: 10.1016/j.plaphy.2023.107726 37167758

[B89] Wang H. R. Du X. R. Zhang Z. Y. Feng F. J. Zhang J. M. (2023). Rhizosphere interface microbiome reassembly by arbuscular mycorrhizal fungi weakens cadmium migration dynamics. iMeta 2, e133. doi: 10.1002/imt2.133 PMC10989832 38868220

[B90] Wang Q. Sun Y. Wang F. Huang P. C. Wang Y. Ruan X.. (2021). Transcriptome and oxylipin profiling joint analysis reveals opposite roles of 9-oxylipins and jasmonic acid in maize resistance to gibberella stalk rot. Front. Plant Sci. 12, 699146. doi: 10.3389/fpls.2021.699146 34557211 PMC8454893

[B91] Wang L. Zhu J. Cui L. Wang Q. Huang W. Yang Q.. (2021). Overexpression of multiple UDP-glycosyltransferase genes involved in sulfoxaflor resistance in *Aphis gossypii* Glover. J. Agric. Food Chem. 69, 5198–5205. doi: 10.1021/acs.jafc.1c00638 33877846

[B92] Wei Q. Lan K. Liu Y. Chen R. Hu T. Zhao S.. (2022). Transcriptome analysis reveals regulation mechanism of methyl jasmonate-induced terpenes biosynthesis in *Curcuma wenyujin* . PloS One 17, e0270309. doi: 10.1371/journal.pone.0270309 35737688 PMC9223393

[B93] Xia Z. Sun B. Wen J. Ma R. Wang F. Wang Y.. (2023). Research progress on metabolomics in the quality evaluation and clinical study of *Panax ginseng.* Biomed. Chromatogr. 37, e5546. doi: 10.1002/bmc.5546 36342761

[B94] Xia Q. Wang Z. Chen X. Dong X. Cheng S. Zhang S. (2023). Effects on the synthesis and accumulation of triterpenes in leaves of *Cyclocarya paliurus* under MeJA treatment. Forests 14, 1735. doi: 10.3390/f14091735

[B95] Xiao H. M. Cai W. J. Ye T. T. Ding J. Feng Y. Q. (2018). Spatio-temporal profiling of abscisic acid, indoleacetic acid and jasmonic acid in single rice seed during seed germination. Anal. Chim. Acta 1031, 119–127. doi: 10.1016/j.aca.2018.05.055 30119729

[B96] Xie W. Hao Z. Yu M. Wu Z. Zhao A. Li J.. (2019). Improved phosphorus nutrition by arbuscular mycorrhizal symbiosis as a key factor facilitating glycyrrhizin and liquiritin accumulation in *Glycyrrhiza uralensis* . Plant Soil 439, 243–257. doi: 10.1007/s11104-018-3861-9

[B97] Xie W. Hao Z. Zhou X. Jiang X. Xu L. Wu S.. (2018). Arbuscular mycorrhiza facilitates the accumulation of glycyrrhizin and liquiritin in *Glycyrrhiza uralensis* under drought stress. Mycorrhiza 28, 285–300. doi: 10.1007/s00572-018-0827-y 29455337

[B98] Yang J. Duan G. Li C. Liu L. Han G. Zhang Y.. (2019). The crosstalks between jasmonic acid and other plant hormone signaling highlight the involvement of jasmonic acid as a core component in plant response to biotic and abiotic stresses. Front. Plant Sci. 10, 1349. doi: 10.3389/fpls.2019.01349 31681397 PMC6813250

[B99] Yang J. L. Hu Z. F. Zhang T. T. Gu A. D. Gong T. Zhu P. (2018). Progress on the studies of the key enzymes of ginsenoside biosynthesis. Molecules 23, 589. doi: 10.3390/molecules23030589 29509695 PMC6017814

[B100] Yang L. Sun Q. Geng B. Shi J. Zhu H. Sun Y.. (2023). Jasmonate biosynthesis enzyme allene oxide cyclase 2 mediates cold tolerance and pathogen resistance. Plant Physiol. 193, 1621–1634. doi: 10.1093/plphys/kiad362 37392433

[B101] Yilmaz A. Karik Ü. (2022). AMF and PGPR enhance yield and secondary metabolite profile of basil (*Ocimum basilicum* L.). Ind. Crops Prod. 176, 114327. doi: 10.1016/j.indcrop.2021.114327

[B102] Yin J. Sun L. Li Y. Xiao J. Wang S. Yang J.. (2020). Functional identification of *BpMYB21* and *BpMYB61* transcription factors responding to MeJA and SA in birch triterpenoid synthesis. BMC Plant Biol. 20, 1–22. doi: 10.1186/s12870-020-02521-1 32787836 PMC7422618

[B103] Yin J. Wang L. Huang Y. Mu Y. Lv S. (2017). Authentication of *Panax ginseng* from different regions. RSC Adv. 7, 55646–55652. doi: 10.1039/C7RA09537F

[B104] Yuan M. L. Zhang M. H. Shi Z. Y. Yang S. Zhang M. G. Wang Z.. (2023). Arbuscular mycorrhizal fungi enhance active ingredients of medicinal plants: a quantitative analysis. Front. Plant Sci. 14, 1276918. doi: 10.3389/fpls.2023.1276918 PMC10623335 37929165

[B105] Yuan J. Zhang W. Sun K. Tang M. J. Chen P. X. Li X.. (2019). Comparative transcriptomics and proteomics of *Atractylodes lancea* in response to endophytic fungus Gilmaniella sp. AL12 reveals regulation in plant metabolism. Front. Microbiol. 10, 1208. doi: 0.3389/fmicb.2019.01208 31191508 10.3389/fmicb.2019.01208 PMC6546907

[B106] Yue P. Jiang Z. Sun Q. Wei R. Yin Y. Xie Z.. (2023). Jasmonate activates a CsMPK6-CsMYC2 module that regulates the expression of β-citraurin biosynthetic genes and fruit coloration in orange (*Citrus sinensis*). Plant Cell 35, 1167–1185. doi: 10.1093/plcell/koac363 36530163 PMC10052374

[B107] Zhang Y. C. Gao S. S. Xue S. An S. H. Zhang K. P. (2021). Disruption of the cytochrome P450 *CYP6BQ7* gene reduces tolerance to plant toxicants in the red flour beetle, *Tribolium castaneum.* Int. J. Biol. Macromol. 172, 263–269. doi: 10.1016/j.ijbiomac.2021.01.054 33453254

[B108] Zhang S. Y. Peng Y. Q. Xiang G. S. Song W. L. Feng L. Jiang X. Y.. (2024). Functional characterization of genes related to triterpene and flavonoid biosynthesis in *Cyclocarya paliurus* . Planta 259, 50. doi: 10.1007/s00425-023-04282-1 38285114

[B109] Zhao Y. Cartabia A. Lalaymia I. Declerck S. (2022). Arbuscular mycorrhizal fungi and production of secondary metabolites in medicinal plants. Mycorrhiza 32, 221–256. doi: 10.1007/s00572-022-01079-0 35556179 PMC9184413

[B110] Zhao M. Lin Y. Wang Y. Li X. Han Y. Wang K.. (2019). Transcriptome analysis identifies strong candidate genes for ginsenoside biosynthesis and reveals its underlying molecular mechanism in *Panax ginseng* CA Meyer. Sci. Rep. 9, 615. doi: 10.1038/s41598-018-36349-5 30679448 PMC6346045

[B111] Zhu L. Hu J. Li R. Liu C. Jiang Y. Liu T.. (2023). Transcriptome-wide integrated analysis of the *PgGT25-04* gene in controlling ginsenoside biosynthesis in *Panax ginseng* . Plants 12, 1980. doi: 10.3390/plants12101980 37653897 PMC10224475

