# Supplementary figures and images for: Improvement of Panax notoginseng saponin accumulation triggered by methyl jasmonate under arbuscular mycorrhizal fungi
Source: Front Plant Sci. 2024 Mar 13;15:1360919. doi: 10.3389/fpls.2024.1360919 (PMC10965624; doi:10.3389/fpls.2024.1360919)

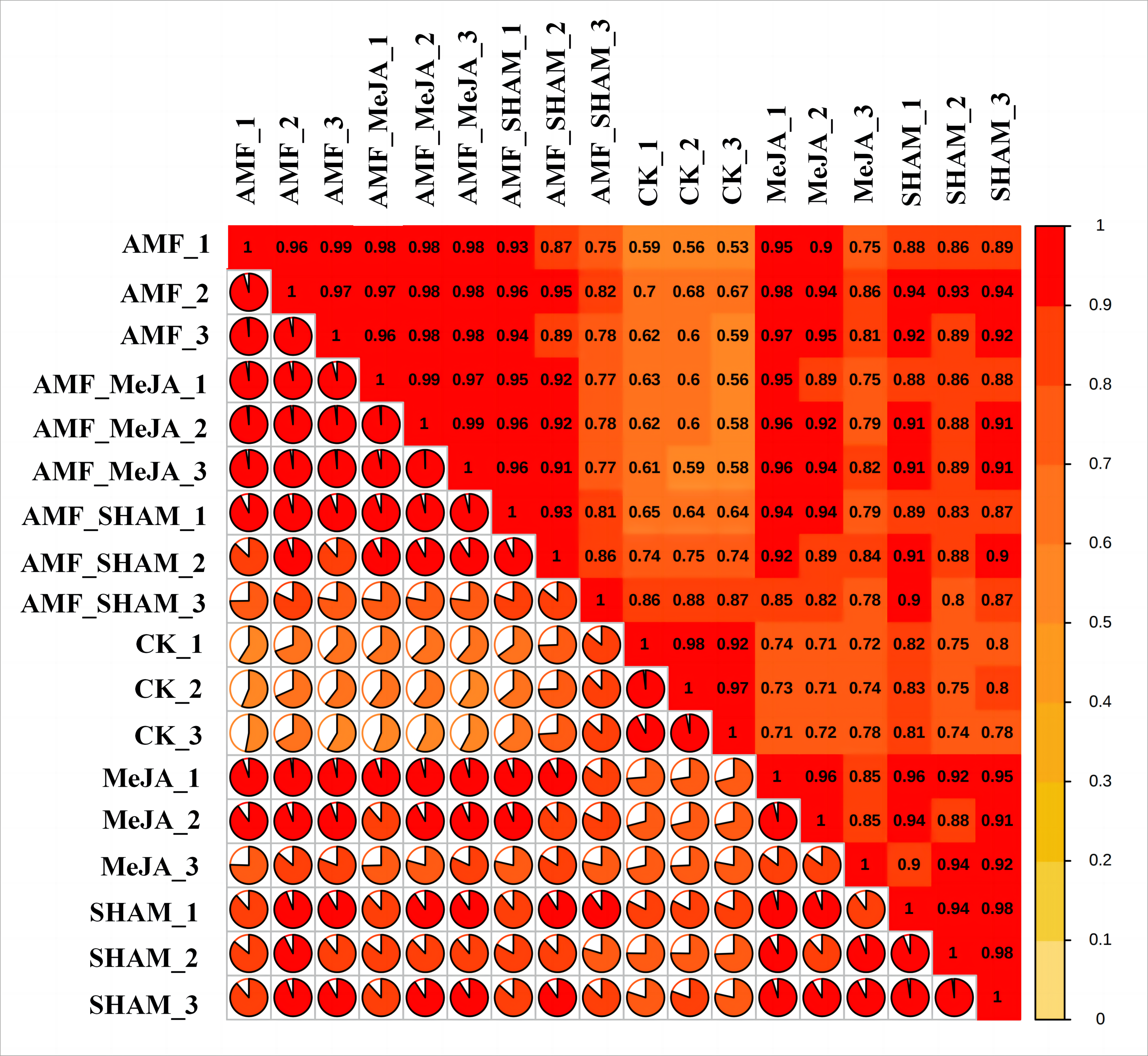

Supplement: Supplementary Figure 1 — Analysis of sample correlation by clustering heat map analysis. Treatment groups of AMF, MeJA, SHAM, AMF-MeJA and AMF-SHAM had strong correlation (R2 ≧ 0.75) between two duplicate samples. However, the correlation between CK and other groups was relative low with a range of 0.53~0.88. [file DataSheet_1.zip › Supplementary Figures 23 12/Figure S1 23 10 11.png]

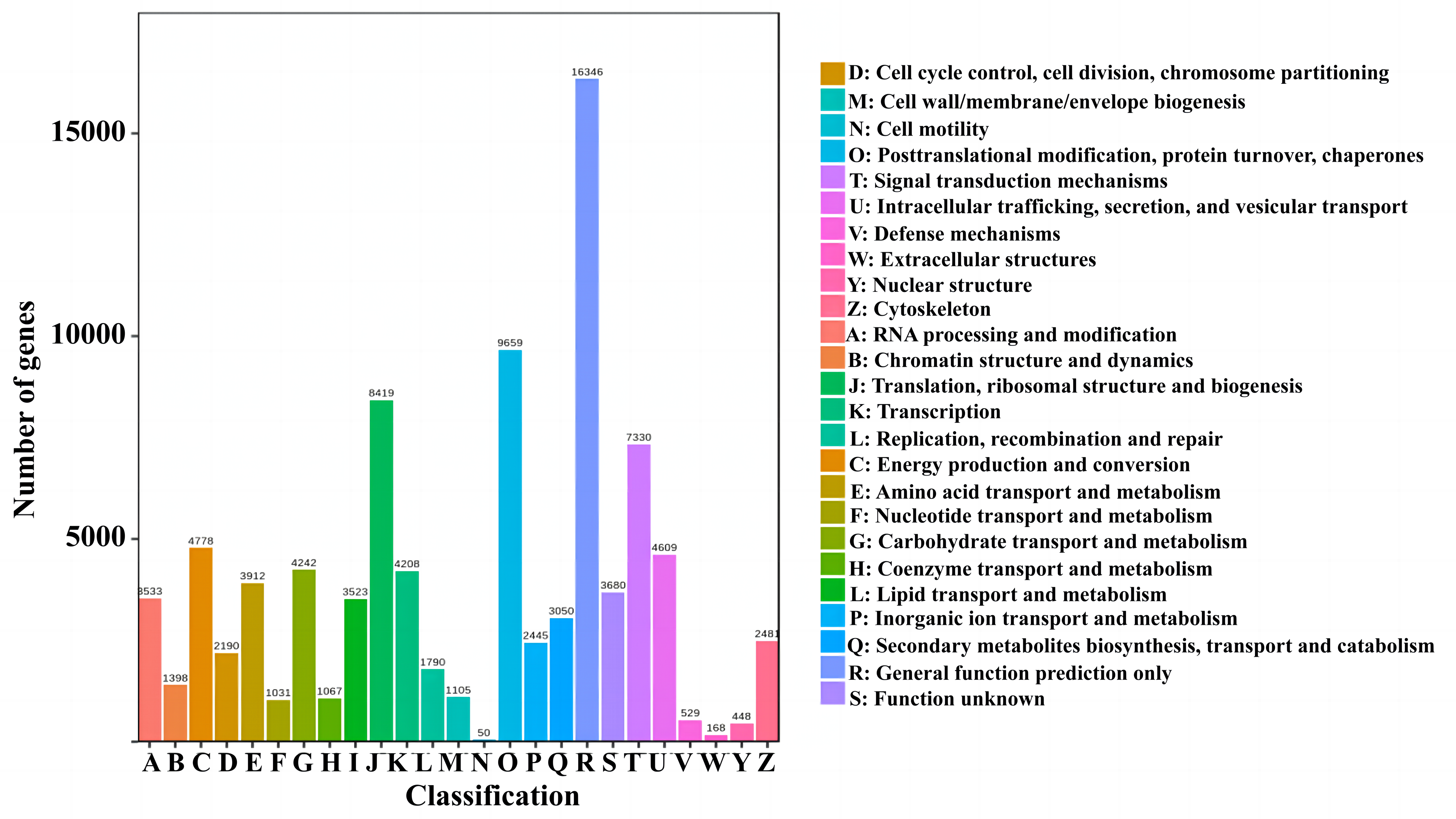

Supplement: Supplementary Figure 1 — Analysis of sample correlation by clustering heat map analysis. Treatment groups of AMF, MeJA, SHAM, AMF-MeJA and AMF-SHAM had strong correlation (R2 ≧ 0.75) between two duplicate samples. However, the correlation between CK and other groups was relative low with a range of 0.53~0.88. [file DataSheet_1.zip › Supplementary Figures 23 12/Figure S2 23 10 11.png]
