# Supplementary material for: Improvement of Panax notoginseng saponin accumulation triggered by methyl jasmonate under arbuscular mycorrhizal fungi
Source: Front Plant Sci. 2024 Mar 13;15:1360919. doi: 10.3389/fpls.2024.1360919 (PMC10965624; doi:10.3389/fpls.2024.1360919)
Supplement: Supplementary file 2 [file DataSheet_2.zip › Supplementary Tables 24 2 25/Supplementary Tab. S1-S7.docx]

Tab. S1 Gradient elution programs for *P. notoginseng* saponin determination

| Time (min) | Acetonitrile (%) | Water (%) |
| --- | --- | --- |
| 0-20 | 20 | 80 |
| 20-60 | 20→46 | 80→54 |
| 60-65 | 46 | 54 |

Tab. S2 Standard curve equations used for *P. notoginseng* saponin determination

| Content | Curve equation | Linear range (mg) | R^2^ |
| --- | --- | --- | --- |
| notoginsenoside R1 | y = 461.08x - 5.7383 | 3.81×10^-4^~3.429×10^-3^ | 1 |
| ginsenoside Rg1 | y = 397.52x - 25.312 | 1.905×10^-3^~1.7145×10^-2^ | 1 |
| ginsenoside Re | y = 455.2x - 15.652 | 1.905×10^-4^~1.7145×10^-3^ | 0.9999 |
| ginsenoside Rb1 | y = 217.42x + 123.39 | 2.286×10^-3^~2.0574×10^-2^ | 0.9941 |
| ginsenoside Rd | y = 447.04x - 4.713 | 3.81×10^-4^~3.429×10^-3^ | 0.9999 |

Tab. S3 Gradient elution programs for JA, MeJA, JA-Ile, OPDA and SA

| Time (min) | Water (containing 0.04% formic acid)% | Acetonitrile (0.04% formic acid) | |
| --- | --- | --- | --- |
| 0 | 95 | | 5 |
| 1 | 95 | | 5 |
| 8 | 5 | | 95 |
| 9 | 5 | | 95 |
| 9.1 | 95 | | 5 |
| 12 | 95 | | 5 |

Tab. S4 Standard curve equations used for the determination of JA, MeJA, JA-Ile, OPDA and SA

| Content | Retention time (min) | Curve equation | Linear range (ng/mL) | R |
| --- | --- | --- | --- | --- |
| JA | 5.83 | y = 0.11124 x + 0.00111 | 0.2~500 | 0.9992 |
| MeJA | 6.93 | y = 0.52790 x - 5.51977e-4 | 0.2~500 | 0.9983 |
| JA-ILE | 6.38 | y = 0.52061 x + 1.73285e-4 | 0.1~500 | 0.9939 |
| OPDA | 7.19 | y = 2.19628 x + 0.05181 | 0.1~500 | 0.9942 |
| SA | 5.09 | y = 0.11660 x + 0.07067 | 0.5~500 | 0.9993 |

Tab. S5 Primer pairs used for qPCR in the confirmatory test of transcriptome

| Name of genes | Sequences Primer (5′-3′) |
| --- | --- |
| *PnCYP450-1* | F:GAGCAAGAAGCCATAATGAGGAC  R:CTAAAAGTGAAGGATAAGATTGAAGCA |
| *PnCYP450-2* | F:ATCATCACCACCTCAAAACGC  R:GGGACGGAACCTAAACGCAT |
| *PnCYP450-4* | F:GAGCAAGAAGCCATAATGAGGAC  R:CTAAAAGTGAAGGATAAGATTGAAGCA |
| *PnGSH-2* | F:TGCCGTGGTTATGATGGTTGTG  R:CATTCTTTCTTTGTAGGATGCTGTTC |
| *PnDXR-2* | F:ATCCTGATGTTTGTCCTCCTTCG  R:ACTCAATCCCATTCCATAATTATACTCT |
| *PnDXR-3* | F:ATCAACTTCTTTCTCTAAGCTCTGGG  R:TCGTCGTGTGGATTCCTCTCG |
| *PnSE-1* | F:CCTGATAAAGCAAGGCAAGAAAT  R:CACGGCAAAGAAGTGGAGAAA |
| *PnSE-2* | F:GCTGATCCTCATCCAACTCCG  R:AGCAACTGTCATTCCCCCG |
| *PnSE-3* | F:GCTTCTCTGTTAGGTTTTCTTTTGG  R:AATGGCGGTGGTTGTGGTC |
| *PnDS-1* | F:CGCAGATAAAAGAAAATCCAAGAGG  R:GCAGTCCGAGACAACGCAAC |
| β-actin (reference genes) | F:TCGGACAACGAGGCAGCACTTT  R:GCTAAAGAGCAGCCAACAGGCC |

Tab. S6 Summary of data output quality of various *P. notoginseng* fibrous root libraries

| Groups | Sample names | Raw reads | Clean data | | | | | | Mapped reads(%) |
| --- | --- | --- | --- | --- | --- | --- | --- | --- | --- |
|  |  |  | Reads | Bases(G) | Errors(%) | Q20(%) | Q30(%) | GC (%) | Total |
| AMF | AMF_1 | 63,743,292 | 61,203,596 | 9.18 | 0.03 | 97.43 | 93.16 | 43.33 | 49,574,070(81.00%) |
|  | AMF_2 | 63,627,440 | 61,794,746 | 9.27 | 0.03 | 97.69 | 93.59 | 43.71 | 47,856,500(77.44%) |
|  | AMF_3 | 71,417,704 | 68,870,576 | 10.33 | 0.03 | 97.72 | 93.71 | 43.09 | 54,920,960(79.75%) |
| AMF-MeJA | AMF-MeJA_1 | 60,788,274 | 58,537,158 | 8.78 | 0.03 | 97.43 | 93.13 | 43.32 | 46,503,042(79.44%) |
|  | AMF-MeJA_2 | 65,076,988 | 62,972,134 | 9.45 | 0.03 | 97.74 | 93.73 | 43.18 | 50,059,062(79.49%) |
|  | AMF-MeJA_3 | 64,034,394 | 61,805,114 | 9.27 | 0.03 | 97.42 | 93.1 | 43.2 | 48,892,528(79.11%) |
| AMF-SHAM | AMF-SHAM_1 | 62,712,138 | 60,264,606 | 9.04 | 0.03 | 97.58 | 93.5 | 42.91 | 45,920,564(76.20%) |
|  | AMF-SHAM_2 | 62,737,054 | 60,358,304 | 9.05 | 0.03 | 97.74 | 93.76 | 43.06 | 47,576,110(78.82%) |
|  | AMF-SHAM_3 | 62,480,186 | 60,021,902 | 9 | 0.03 | 97.67 | 93.57 | 43.96 | 48,091,140(80. 12%) |
| MeJA | MeJA_1 | 65,219,420 | 60,557,902 | 9.08 | 0.03 | 97.77 | 93.85 | 43.52 | 48,291,990(79.75%) |
|  | MeJA_2 | 63,493,248 | 61,196,596 | 9.18 | 0.03 | 97.64 | 93.49 | 43.28 | 48,835,548(79.80%) |
|  | MeJA_3 | 60,522,188 | 59,082,372 | 8.86 | 0.03 | 97.76 | 93.78 | 43.21 | 46,969,414(79.50%) |
| SHAM | SHAM_1 | 58,022,316 | 56,378,686 | 8.46 | 0.03 | 97.82 | 93.98 | 43.27 | 44,531,402(78.99%) |
|  | SHAM_2 | 64,825,458 | 61,237,844 | 9.19 | 0.03 | 97.96 | 94.36 | 43.22 | 49,449,072(80.75%) |
|  | SHAM_3 | 74,971,714 | 68,616,856 | 10.29 | 0.03 | 97.84 | 94.05 | 43.1 | 54,672,574(79.68%) |
| CK | CK_1 | 68,786,130 | 66,363,142 | 9.95 | 0.03 | 97.78 | 93.87 | 43.5 | 52,462,416(79.05%) |
|  | CK_2 | 60,412,464 | 58,237,308 | 8.74 | 0.03 | 97.84 | 94.05 | 43.17 | 45,760,516(78.58%) |
|  | CK_3 | 62,845,858 | 60,684,326 | 9.1 | 0.03 | 97.78 | 93.83 | 43.46 | 48,441,976(79.83%) |

Tab. S7 Functional annotations of *P. notoginseng* unigenes in five protein databases

| Database | Number of Unigenes | Percentage (%) |
| --- | --- | --- |
| Annotated in Nr | 124,110 | 48.19 |
| Annotated in KEGG | 94,304 | 36.62 |
| Annotated in Pfam | 77,830 | 30.22 |
| Annotated in Trembl | 124,298 | 48.26 |
| Annotated in Swissprot | 84,972 | 32.99 |
| Annotated in at least one Database | 135,099 | 52.46 |
| Total Unigenes | 257,548 | 100 |
